# Carbon Black Nanoparticle–PP Fiber Interfacial Engineering for Piezoresistive Self-Sensing Cementitious Nanocomposites

**DOI:** 10.3390/nano16160999

**Published:** 2026-08-13

**Authors:** Xianyang Fu, Yongchun Hao

**Affiliations:** School of Architecture and Art, Central South University, Changsha 410075, China; 241312013@csu.edu.cn

**Keywords:** carbon black nanoparticles, polypropylene fiber, cement-based sensor, interfacial engineering, piezoresistive cement nanocomposite

## Abstract

Carbon black (CB) nanoparticles (~20 nm) offer high specific surface area and conductivity for self-sensing cementitious composites, but strong interparticle van der Waals forces drive agglomeration in alkaline pore solutions, limiting sensing reliability. This study introduces a nanoscale interfacial engineering strategy in which CB nanoparticles are adsorbed onto polypropylene (PP) fiber surfaces as spatially organized conductive elements, with EDS evidence of enhanced hydrate coverage at the fiber–matrix interface. Three CB dosages (0.5%, 1.0%, and 1.5% by binder mass) with 0.5% PP fiber were investigated. Nanoparticle coating and interfacial micro-structure were characterized by SEM-EDS, while FTIR was used to verify that the fiber backbone remained chemically unmodified; piezoresistive response and durability were assessed via cyclic compression, DIC, and hygrothermal cycling. The 1.0% CB nanocomposite lies within the effective percolation window (~0.9–1.2%), showing high linearity, a stable gauge factor (~100), and distinct FCR acceleration for early-warning sensing. The 1.5% CB composite yields higher sensitivity but scattered responses due to nanoparticle clustering; 0.5% CB remains below the percolation threshold with a discontinuous network. After 60 hygrothermal cycles, the 1.0% nanocomposite retains >93% of its gauge factor with minimal resistance drift. The nano-engineered CB–PP fiber architecture offers a scalable route integrating crack bridging, percolation networking, and durable self-sensing in cementitious nanocomposites for structural health monitoring.

## 1. Introduction

Earthquakes, growing traffic loads, and aging infrastructure expose transportation systems—railways, bridges, and tunnels—to increasingly complex service-life risks. Conventional inspection relies on visual walks, discrete sensors, or periodic testing. These approaches struggle to track damage as it evolves from micro-crack initiation to macro-fracture within concrete. Self-sensing cementitious composites offer a promising alternative. Embedded directly in the host structure, they deform in concert with the matrix and enable low-cost, durable monitoring over broad spatial coverage [1,2,3].

At the heart of self-sensing cementitious materials lies a conductive network responsive to stress, strain, and crack propagation. Carbon-based fillers—carbon fibers, carbon nanotubes, graphene, and carbon black—have been widely adopted to enhance the piezoresistive response of cement matrices [4,5,6,7]. Among these, carbon black (CB) nanoparticles (~20 nm) stand out for their nanoscale conductivity, high specific surface area, low cost, and ready availability, making them attractive for field-scale deployment [8,9,10]. Yet, CB has a high specific surface area and surface energy. In the alkaline, ion-rich pore solution of cement, particles tend to agglomerate, yielding uneven networks, signal drift, and degraded mechanical performance [11,12,13].

Polypropylene (PP) fibers, by contrast, suppress crack growth through bridging and pull-out energy dissipation, improving toughness and cracking resistance [14,15,16]. They are, however, electrically inert and cannot serve a sensing role on their own. Prior work has blended CB, CNTs, or hybrid fillers directly into the matrix to lower resistivity and raise sensitivity. Most systems, though, encounter two persistent limitations. At low dosages, conductive paths remain discontinuous, and the signal is unstable. At high dosages, filler agglomeration and interfacial defects intensify hysteresis, drift, and abrupt damage transitions [17,18,19,20,21].

The scientific question here is not which CB dosage optimizes conductivity. It is how to strike an interpretable balance among fiber reinforcement, electrical connectivity, and long-term signal stability [22,23,24]. To address this, we exploit an interfacial engineering strategy rooted in the hydrophobic affinity between CB and PP fibers. CB particles preferentially adsorb onto the fiber surface in a uniform, mechanically stable layer, forming an integrated reinforcement–sensing network. This converts CB from randomly dispersed, agglomeration-prone particles into continuous conductive elements aligned along each fiber. The expected outcome is a lower percolation threshold and improved signal reliability.

Guided by this rationale, we systematically characterize CB-coated PP fiber sensors at 0.5%, 1.0%, and 1.5% CB loading. The investigation spans interfacial evidence, percolation behavior, cyclic compression response, flexural crack monitoring, splitting-tensile early warning, damage-field evolution, and hygrothermal cycling durability [25,26,27,28]. Our central hypothesis posits that 1.0% CB lies near the effective percolation window where the network transitions from discontinuous to continuous. This composition should yield an optimal balance of conductivity, linear sensing, and stability. At 1.5% CB, stronger electrical signals are expected, yet agglomeration and localized damage events may compromise long-term reliability [29,30].

## 2. Materials and Methods

### 2.1. Raw Materials

General-purpose Portland cement conforming to AS 3972-2010 [31] was used; it consists primarily of clinker, gypsum, and a minor fraction of limestone. Silica fume replaced 5% of the cementitious material by mass to enhance packing density and refine the pore structure. The CB particles had a mean diameter of ~20 nm, a volume resistivity below 0.4 Ω·cm, a DBP absorption of 280 mL/100 g, and a specific surface area of 254 m^2^/g. PP fibers measured 9 mm in length and 18–48 μm in diameter, with a tensile strength exceeding 486 MPa, an elastic modulus above 4.8 GPa, and an elongation at break greater than 15%. A polycarboxylate-based superplasticizer was added to control the workability of the fresh mix.

### 2.2. Mix Proportions

Three CB dosages were investigated—0.5%, 1.0%, and 1.5% by mass of cementitious material—designated 05CB, 10CB, and 15CB, respectively. The PP fiber content was held constant at 0.5% by binder mass, and the water-to-binder ratio was fixed at 0.45. As CB loading rose, the superplasticizer dosage was adjusted to 0.6%, 0.8%, and 1.0% to offset the flowability loss induced by CB and ensure consistent specimen quality.

### 2.3. Definition and Normalization of Derived Performance Indices

To improve the reproducibility of the quantitative comparisons among different CB dosages, the derived performance metrics used in the subsequent analysis were explicitly defined prior to interpretation of the results. These metrics include the EDS-derived interfacial composition ratio, moisture stability index, CB agglomeration index, cyclic reversibility, and overall stability score.

The fiber–matrix interfacial composition was quantified directly from the EDS atomic fractions measured within identical interfacial regions of interest (ROIs). The C/(Ca + Si) interfacial composition ratio was calculated as follows:

(1) $$R_{int}=\frac{X_{C}}{X_{Ca}+X_{Si}}$$

where $X_{C}$, $X_{Ca}$, and $X_{Si}$ are the EDS atomic fractions of C, Ca, and Si, respectively. $R_{int}$ is reported as a directly measured compositional descriptor rather than as a normalized measure of interfacial bond strength. Its interpretation was therefore combined with the Si signal and the spatial continuity of Ca–Si-containing hydration products observed by EDS mapping.

The moisture stability index was introduced to quantify the magnitude of the resistivity variation induced by moisture cycling and was defined as follows:

(2) $$I_{M}=\frac{\min\left({\bar{\rho }}_{dry}{\bar{\rho }}_{moist}\right)}{\max\left({\bar{\rho }}_{dry}{\bar{\rho }}_{moist}\right)}$$

where ${\bar{\rho }}_{dry}$ and ${\bar{\rho }}_{moist}$ denote the mean electrical resistivities measured under the dry and moisture-cycled conditions, respectively. The index satisfies 0 < $I_{M}\leq$ 1. A value approaching unity represents a smaller moisture-induced variation in electrical resistivity and therefore greater environmental stability of the conductive network.

CB agglomeration was quantified from SEM/EDS images using identical segmentation criteria for all specimens. A CB-rich connected region exceeding the predefined equivalent-diameter threshold was classified as an agglomerated region. The equivalent diameter of a connected region with projected area AAA was determined from

(3) $$d_{eq}=2\sqrt{\frac{A}{\pi }}$$

The agglomeration index was then calculated from the pooled image area as

(4) $$I_{agg}=\frac{\sum_{m=1}^{M}A_{agg,m}}{\sum_{m=1}^{M}A_{CB,m}}$$

where $A_{agg,m}$ represents the total area classified as CB agglomerates in the m-th field, $A_{CB,m}$ represents the total CB-rich area in the same field, and M is the total number of analyzed fields. $I_{agg}$ ranges from 0 to 1, with increasing values indicating that a larger fraction of the CB-rich phase occurs in agglomerated form.

Cyclic reversibility was evaluated from the residual FCR after each loading–unloading cycle. For the k-th cycle, the recovery index was calculated as

(5) $$r_{k}=1-\frac{\left|{FCR}_{k,end}-{FCR}_{k,start}\right|}{\left|{FCR}_{k,peak}-{FCR}_{k,start}\right|}$$

To maintain a physically meaningful normalized range, the cyclic reversibility was defined as

(6) $$R_{v}=\frac{1}{N}\sum_{k=1}^{N}\max[0,\min(1,r_{k})]$$

where N is the total number of loading–unloading cycles and ${FCR}_{k,start}$, ${FCR}_{k,peak}$, and ${FCR}_{k,end}$ are the FCR values at the beginning of loading, at maximum applied stress, and after complete unloading of the k-th cycle, respectively. $R_{v}=1$ represents complete recovery of the electrical response after unloading, whereas a decreasing value indicates increasing irreversible residual response.

For the integrated sensitivity–stability evaluation, the FCR–stress linearity component was represented by the coefficient of determination $R_{FCR-\sigma }^{2}$ obtained from linear regression. This quantity is distinguished from the maximum linearity error used separately to characterize deviation from the fitted response. The overall stability score was calculated by assigning equal weights to FCR–stress linearity and cyclic reversibility:

(7) $$S_{stab}=\frac{R_{FCR-\sigma }^{2}+R_{v}}{2}$$

where $0\leq I_{M}<$ 1. A larger stability score indicates a better combination of linear stress tracking and recovery stability under cyclic loading.

### 2.4. Preparation of CB-Coated PP Fiber Cement-Based Sensors

**Figure 1:**
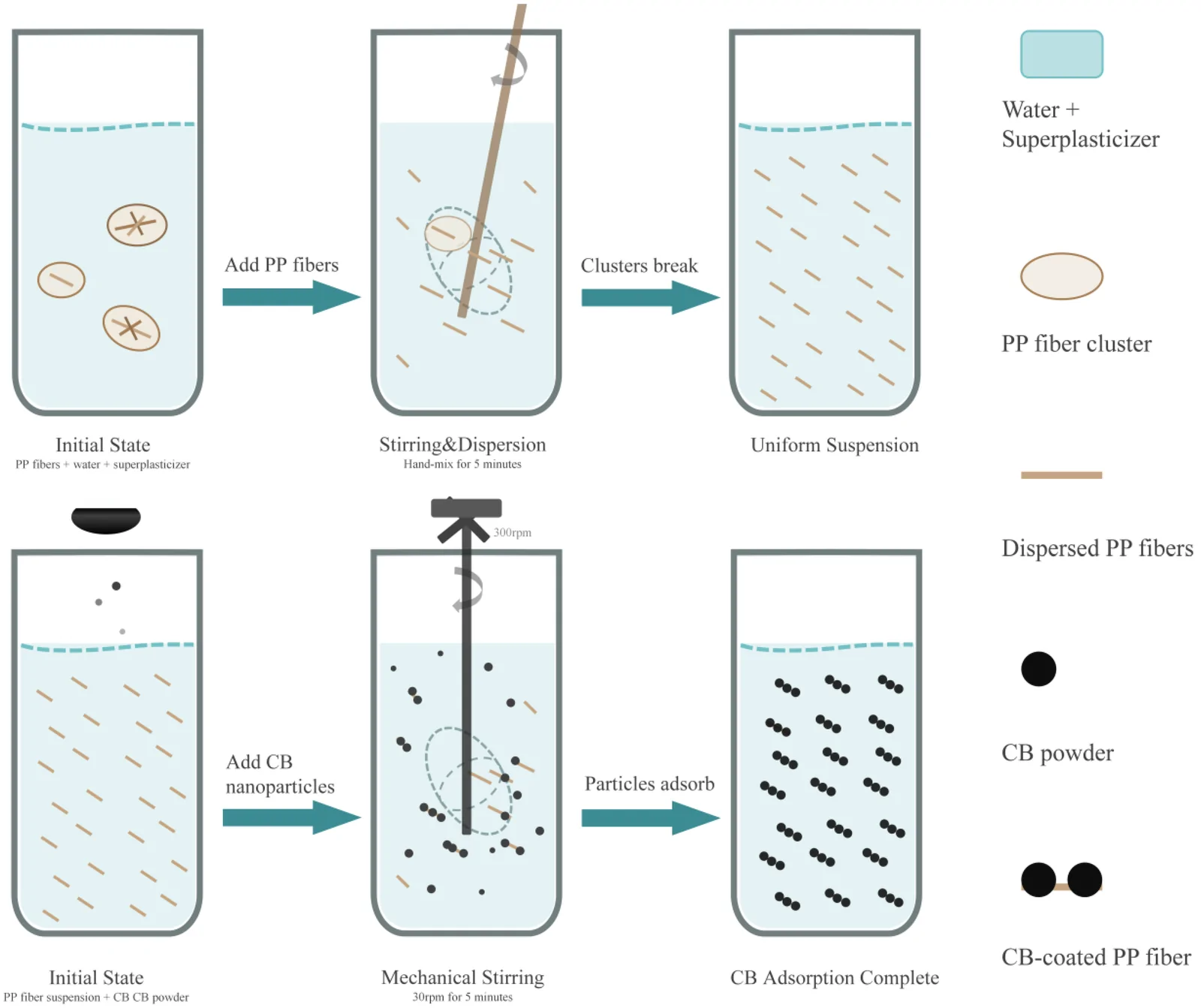
Schematic illustration of CB coating process on PP fiber surface via hydrophobic interaction.

The critical objective during specimen preparation was to direct CB onto the PP fiber surface rather than allowing random agglomeration in the matrix. The procedure comprised two sequential stages. PP fibers were introduced into a solution of water and superplasticizer, then stirred manually for 5 min to separate the fiber bundles into a uniform suspension. Pre-weighed CB was then added gradually to this suspension and mechanically stirred at 300 rpm for 5 min, allowing the particles to wet and adsorb onto the fiber surface. The CB-coating procedure is schematically illustrated in Figure 1.

The suspension was further subjected to a 40 kHz ultrasonic bath for 1 h. The bath water was refreshed every 10 min to prevent heat buildup from re-agglomerating the CB.

**Figure 2:**
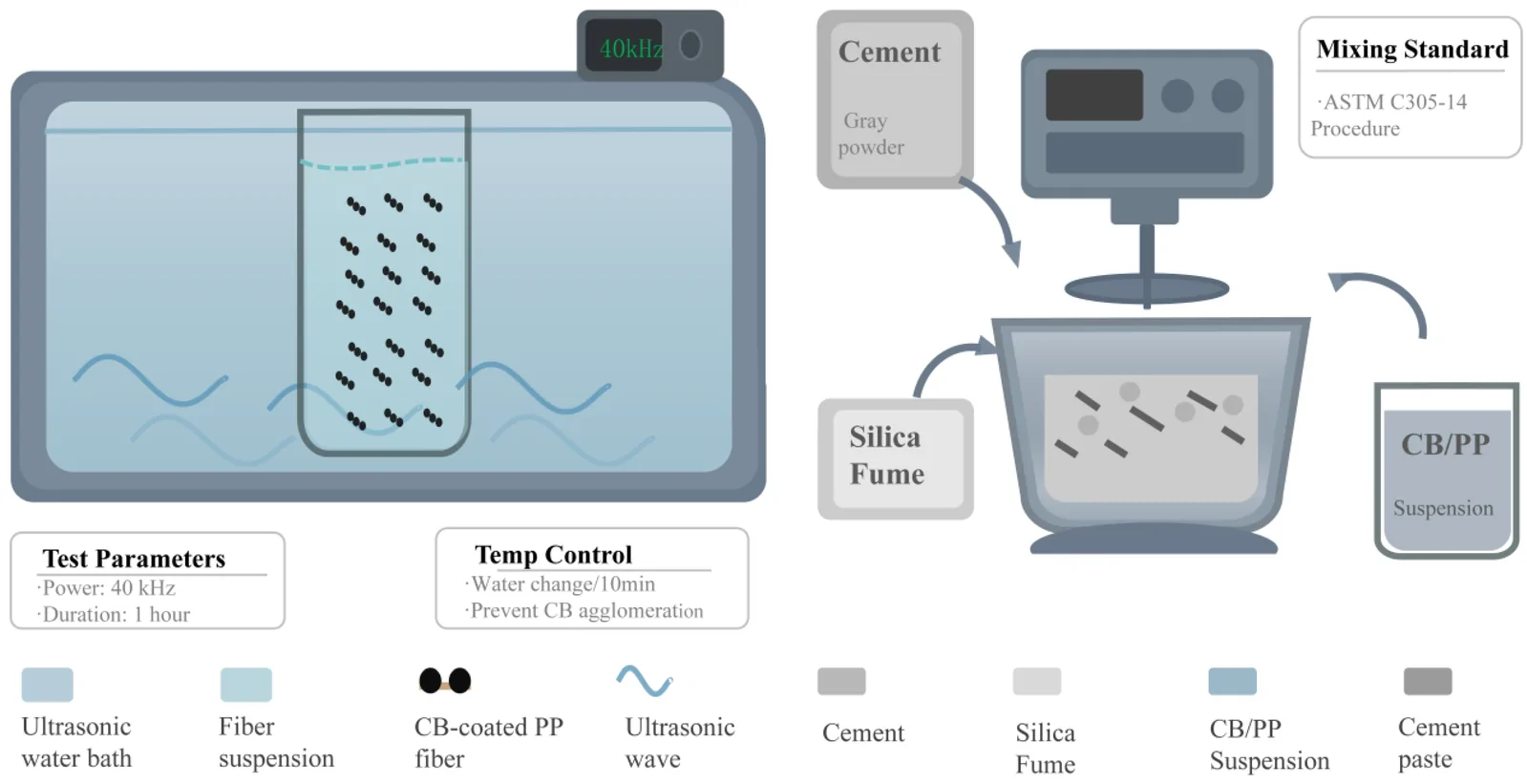
Preparation procedure of CB/PP fiber cement paste, including ultrasonic treatment and standard mixing. Arrows indicate the sequential preparation steps; the wavy line denotes the ultrasonic water bath, and the material symbols correspond to those identified in the legend.

Throughout this process, the hydrophobic affinity between CB and PP drives particle enrichment and coating along the fiber surface. The overall preparation and mixing procedure is summarized in Figure 2.

**Figure 3:**
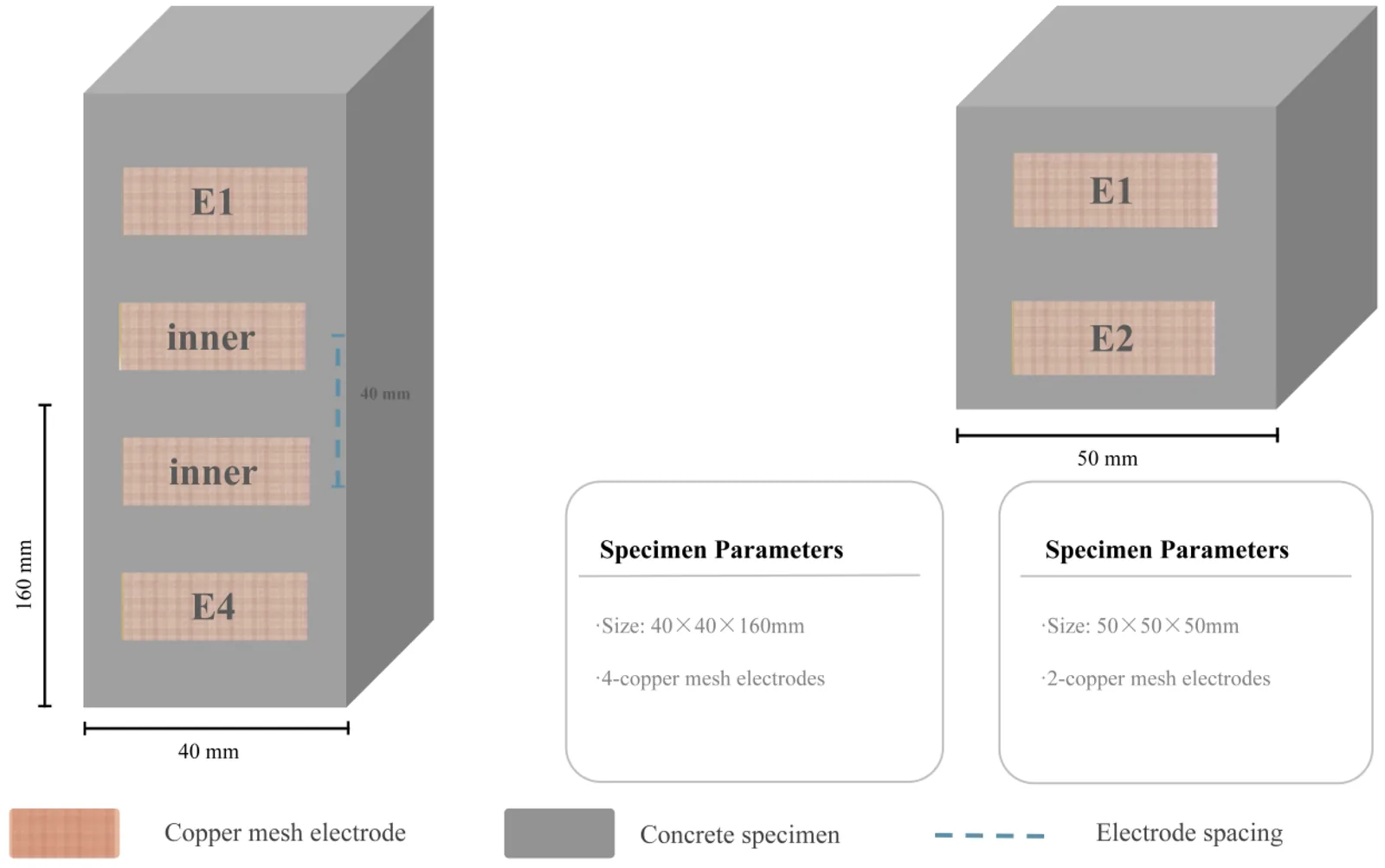
Specimen geometry and electrode configuration: prismatic four-probe setup and cubic two-probe setup.

Two specimen geometries were cast: 40 mm × 40 mm × 160 mm prisms and 50 mm × 50 mm × 50 mm cubes. Each prism contained four embedded copper-mesh electrodes with an inner-electrode spacing of 40 mm; each cube contained two, spaced identically. After casting, specimens were vibration-compacted and sealed with plastic film to limit early-age moisture loss. The specimen geometries and electrode configurations are shown in Figure 3.

**Figure 4:**
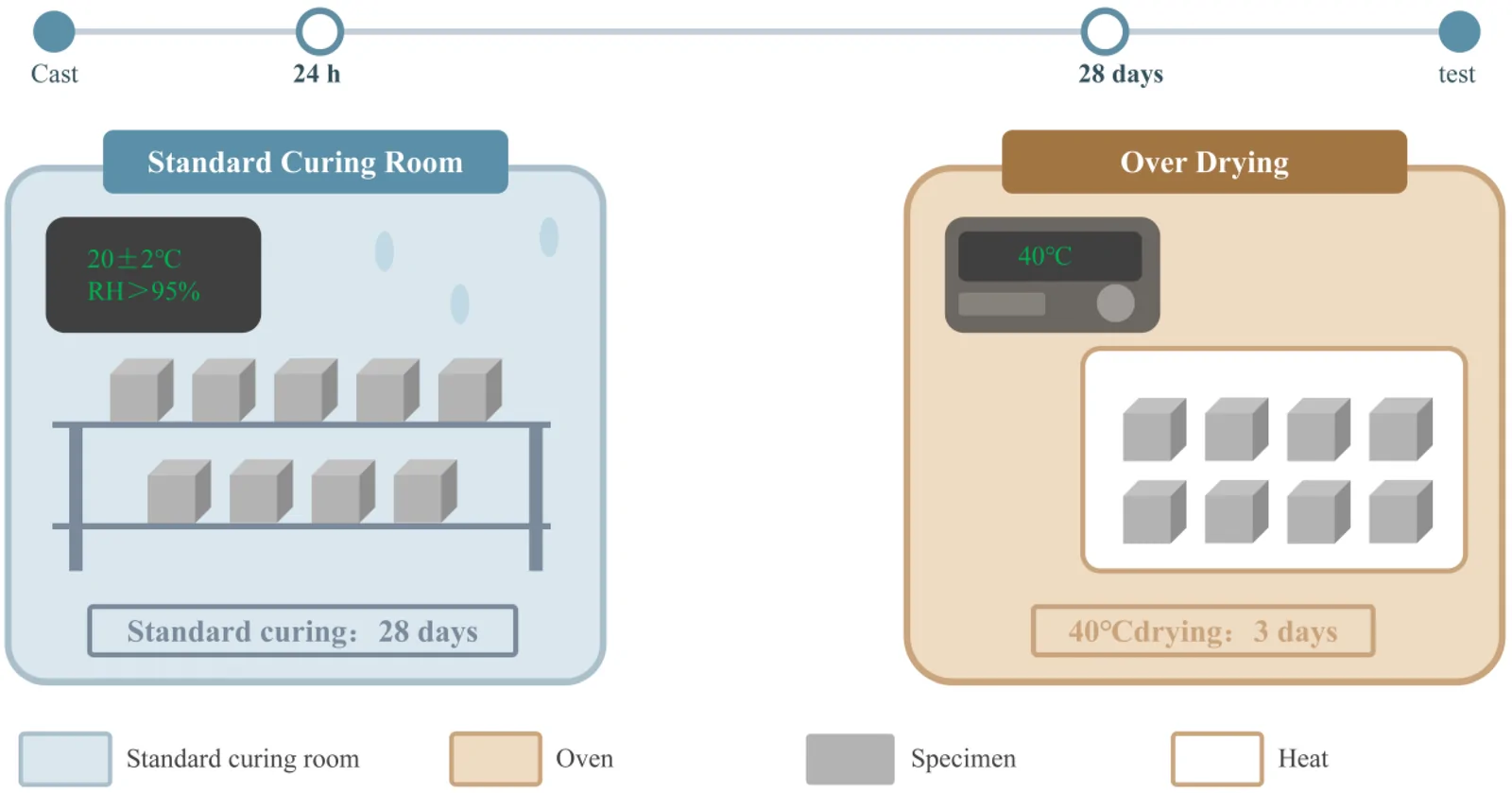
Flowchart of specimen curing and drying pretreatment. Filled circles indicate the casting and testing points, whereas open circles indicate the 24 h demolding and 28 d curing milestones.

Specimens remained in a curing room at 20 ± 2 °C and above 95% RH for 24 h before demolding, then cured until 28 d. Those designated for dry-state electrical testing were oven-dried at 40 °C for 3 d prior to measurement. The curing and drying pretreatment protocol is summarized in Figure 4.

### 2.5. Microstructural and Interfacial Characterization

CB distribution on the PP fiber surface and within the cement matrix was examined using a Zeiss EVO LS15 SEM (Carl Zeiss AG, Oberkochen, Germany) equipped with an EDS detector. Samples (5 mm × 5 mm × 2 mm) were extracted from fractured specimen interiors, vacuum-dried, and gold-sputtered before imaging. EDS mapping targeted C, Ca, Si, O, and Al to semi-quantitatively assess CB coating efficiency, the fiber–matrix interfacial transition zone, and hydration product deposition around the fibers.

**Figure 5:**
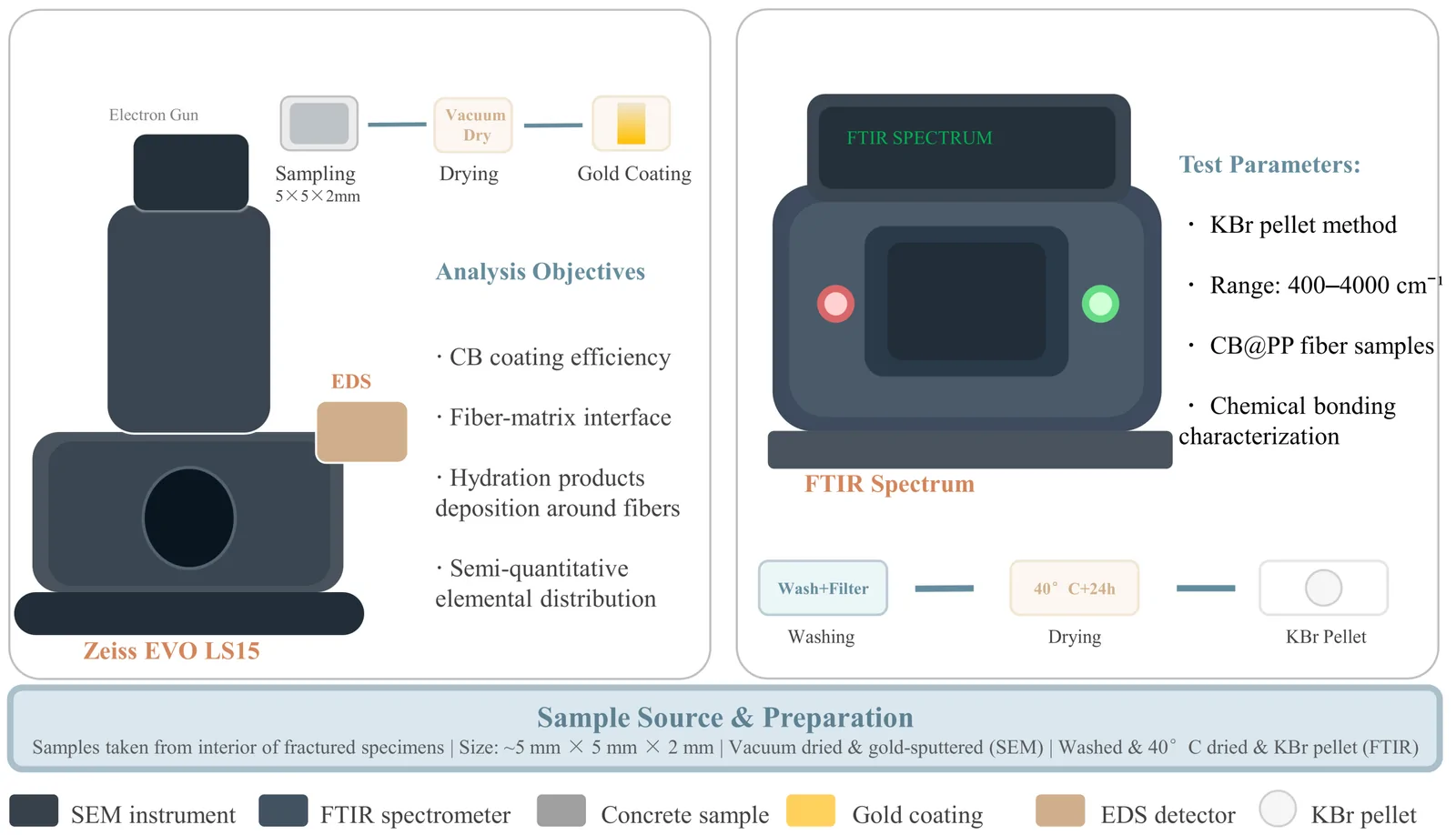
Microstructural and interfacial characterization methods: SEM-EDS mapping and FTIR.

Fourier-transform infrared spectroscopy (FTIR) was used to analyze CB-coated PP fibers extracted after ultrasonication. Samples were repeatedly washed, settled, and filtered with deionized water to remove loosely physisorbed particles, then dried at 40 °C for 24 h. Spectra were acquired using the KBr pellet method over 400–4000 cm^−1^ at a resolution of 4 cm^−1^. The SEM-EDS and FTIR characterization workflow is summarized in Figure 5.

### 2.6. Electrical Measurement

**Figure 6:**
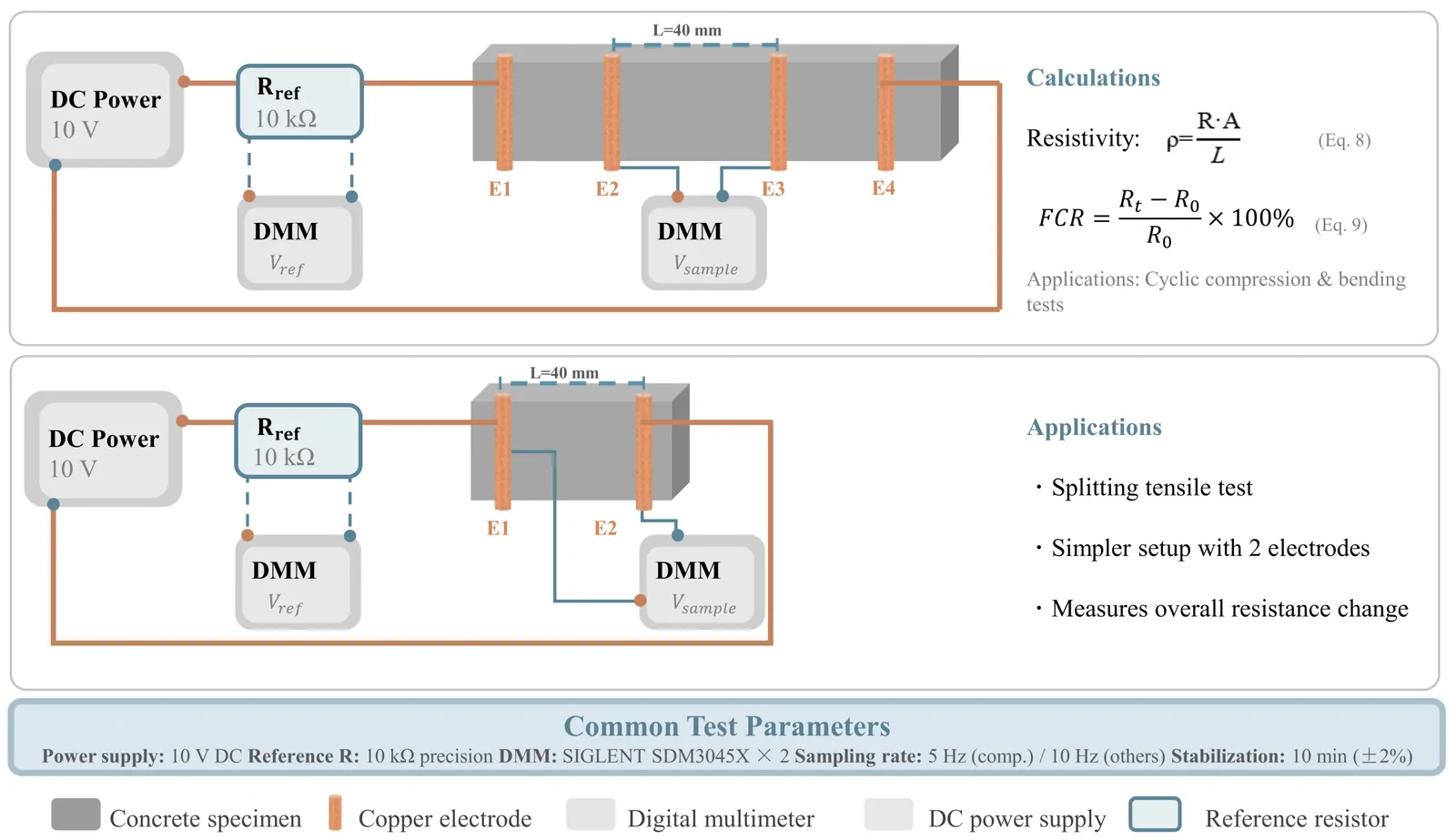
Circuit diagrams for the four-probe and two-probe electrical measurements.

Resistance was measured by the four-probe method for cyclic compression and notched bending on prisms, and by the two-probe method for splitting tension on cubes. The circuit placed a 10 V DC supply, a 10 kΩ precision reference resistor, and the specimen in series. Two SIGLENT SDM3045X multimeters (SIGLENT Technologies Co., Ltd., Shenzhen, China) simultaneously logged the voltage drops across the reference resistor and the specimen. Sampling rates were set at 5 Hz for cyclic compression and 10 Hz for bending and splitting tests. Before each test, specimens were energized for 10 min until resistance fluctuations fell below ±2%. The four-probe and two-probe electrical measurement circuits are shown in Figure 6.

Volume resistivity was calculated using Equation (8) as follows:

(8) $$\rho \;=\;\frac{R\cdot A}{L}$$

where R is the initial specimen resistance (Ω), A is the effective conductive cross-sectional area (m^2^), and L is the electrode spacing (m).

The fractional change in resistivity (FCR) was determined from Equation (9) as follows:

(9) $$\mathrm{FCR}\;=\;\frac{\rho_{t}-\rho_{0}}{\rho_{0}}\times 100\%\;=\;\frac{R_{t}-R_{0}}{R_{0}}\times 100\%\;$$

where $\rho_{t}$ and $R_{t}$ are the resistivity and resistance at time t, and $\rho_{0}$ and $R_{0}$ are their initial values at the onset of loading.

### 2.7. Cyclic Compression Test

**Figure 7:**
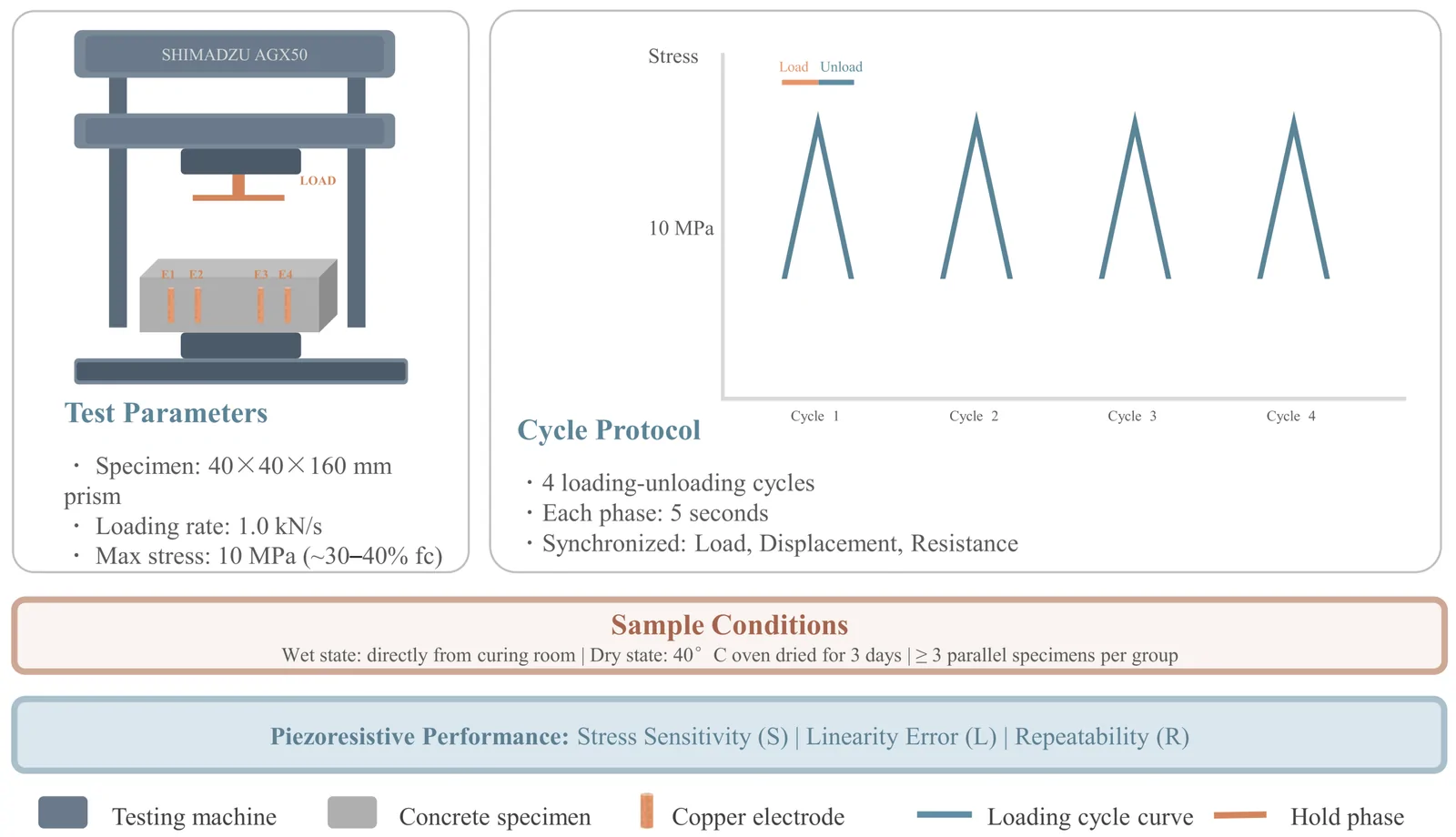
Cyclic compression test setup and loading protocol.

Cyclic compression tests were conducted on a SHIMADZU AGX50 universal testing machine (Shimadzu Corporation, Kyoto, Japan). Prisms (40 mm × 40 mm × 160 mm) were centered between the loading platens and loaded at 1.0 kN/s to 10 MPa. This stress level corresponds to roughly 30–40% of the estimated compressive strength, ensuring a reversible elastic regime. Each specimen underwent four load–unload cycles with 5 s per stage. Load, displacement, and resistance were recorded synchronously throughout. Wet specimens were tested directly from the curing room; dry specimens were oven-dried at 40 °C for 3 d beforehand. A minimum of three replicates was tested per group. The cyclic compression test setup and loading protocol are shown in Figure 7.

Piezoresistive performance was evaluated by three metrics: stress sensitivity, linearity error, and repeatability. Stress sensitivity S is the slope of the linear fit to the FCR–stress curve. Linearity error L quantifies the maximum deviation of measured FCR from that fit. Repeatability R characterizes the consistency of FCR across multiple cycles at the same stress level.

### 2.8. Notched Three-Point Bending Test

**Figure 8:**
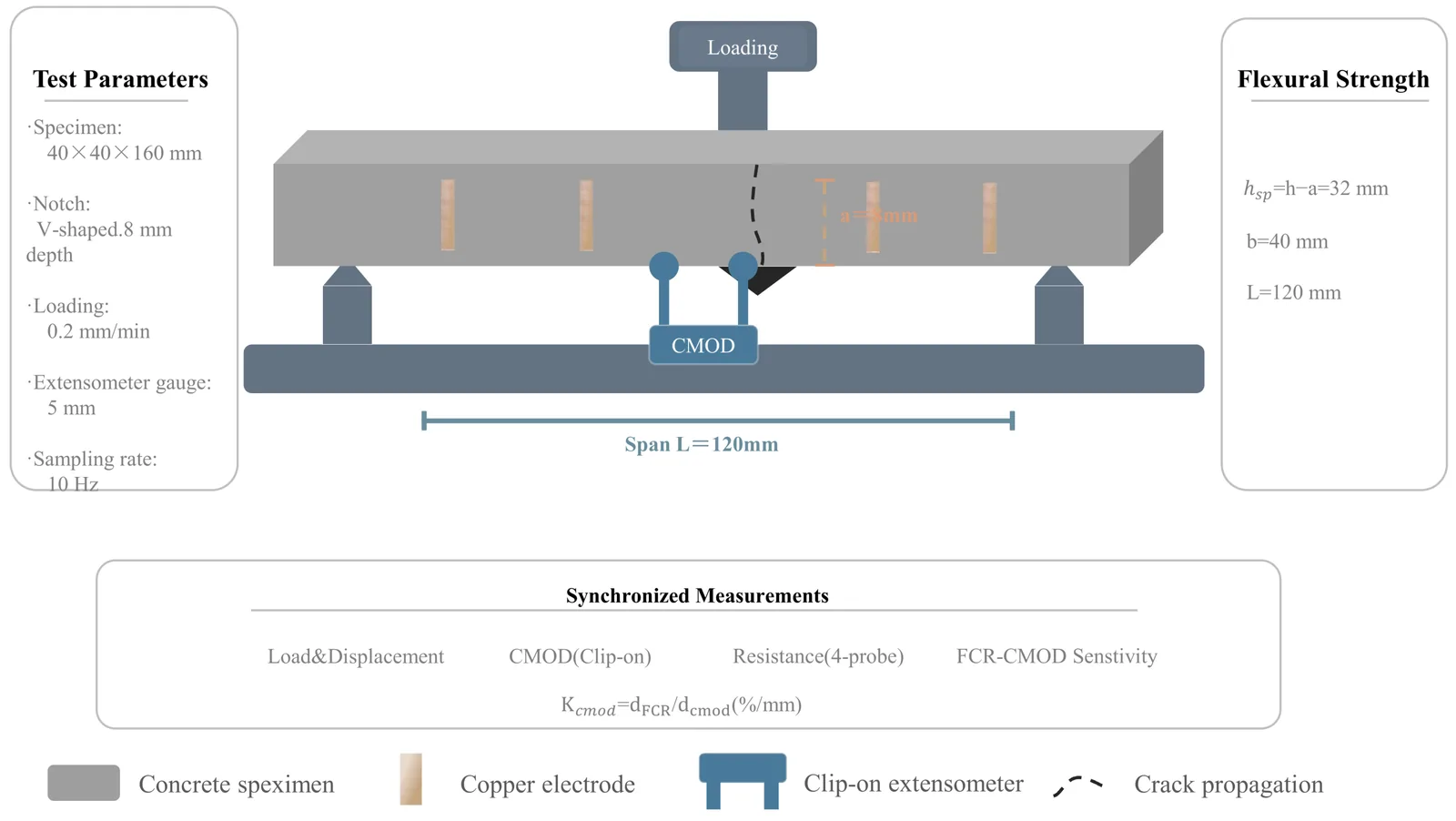
Notched three-point bending test setup with CMOD monitoring.

A V-notch was cut at the mid-span soffit of each prism (40 mm × 40 mm × 160 mm) with a span of 120 mm, a notch depth of 8 mm, and a width of approximately 1.5 mm. Loading was displacement-controlled at a rate of 0.2 mm/min. A clip-on extensometer mounted at the notch mouth recorded the crack mouth opening displacement (CMOD) in real time, while load, displacement, CMOD, and resistance were logged synchronously. The notched three-point bending setup with CMOD monitoring is shown in Figure 8.

The flexural strength $f_{m}$ was calculated following CECS 13-2009 [32]:

(10) $$f_{m}\;=\;\frac{3F_{max}L}{2bh_{\mathrm{sp}}^{2}}$$

where $F_{max}$ is the peak load (N), L is the span (120 mm), b is the specimen width (40 mm), and $h_{sp}$ is the effective height after deducting the notch (32 mm). CMOD sensitivity, defined as $K_{cmod}=\frac{d_{FCR}}{d_{cmod}}$, was extracted from the FCR–CMOD curve to evaluate the electrical response of the conductive network to crack opening.

### 2.9. Splitting Tensile Test

**Figure 9:**
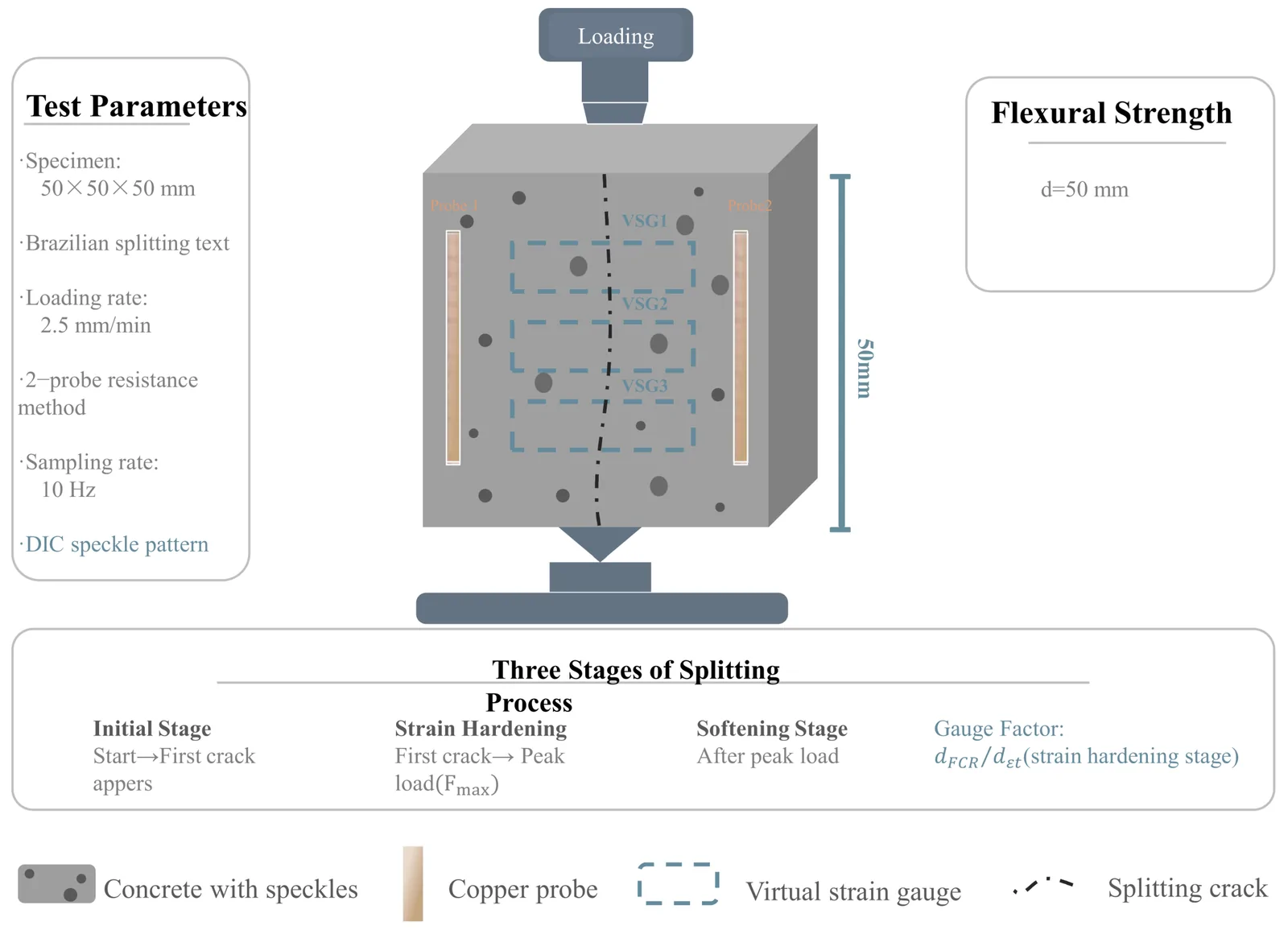
Brazilian splitting tensile test setup with DIC tracking.

Brazilian splitting tests were performed on 50 mm × 50 mm × 50 mm cubes at a loading rate of 2.5 mm/min. A random speckle pattern was applied to the front face for DIC analysis, while resistance was monitored via the two-probe method. Horizontal tensile strain was averaged from three virtual gauge lengths of 15 mm oriented horizontally across the front face. The Brazilian splitting tensile test setup and DIC tracking configuration are shown in Figure 9.

The splitting tensile strength was obtained from Equation (11) as follows:

(11) $$f_{\mathrm{ts}}=\frac{2F_{\max}}{\pi d^{2}}$$

where $F_{\max}$ is the peak load (N) and d is the cube side length (50 mm). Based on the load–displacement curve and DIC strain field, the splitting process was divided into an initial stage, a strain-hardening stage, and a softening stage; the FCR–tensile strain relationship was analyzed for each. The gauge factor during strain hardening $(\mathrm{GF}\;=\;d_{\mathrm{FCR}}/d_{\varepsilon t})$ served to evaluate the sensing sensitivity to tensile deformation.

### 2.10. Digital Image Correlation

DIC was employed to track the full-field displacement and strain evolution during both notched bending and splitting tension. The imaging system comprised a FLIR Grasshopper3 GS3-U3-23S6M-C camera (Teledyne FLIR Integrated Imaging Solutions, Richmond, BC, Canada, 1920 × 1200 pixels), a Schneider Kreuznach 50 mm f/2.8 lens (Jos. Schneider Optische Werke GmbH, Bad Kreuznach, Germany), and an LED ring light. Speckle patterns were prepared with white primer and random black dots. Displacement and strain fields were computed using VIC-2D 2009 (Correlated Solutions, Inc., Irmo, SC, USA) with a subset size of 29 × 29 pixels and a step size of 5 pixels.

**Figure 10:**
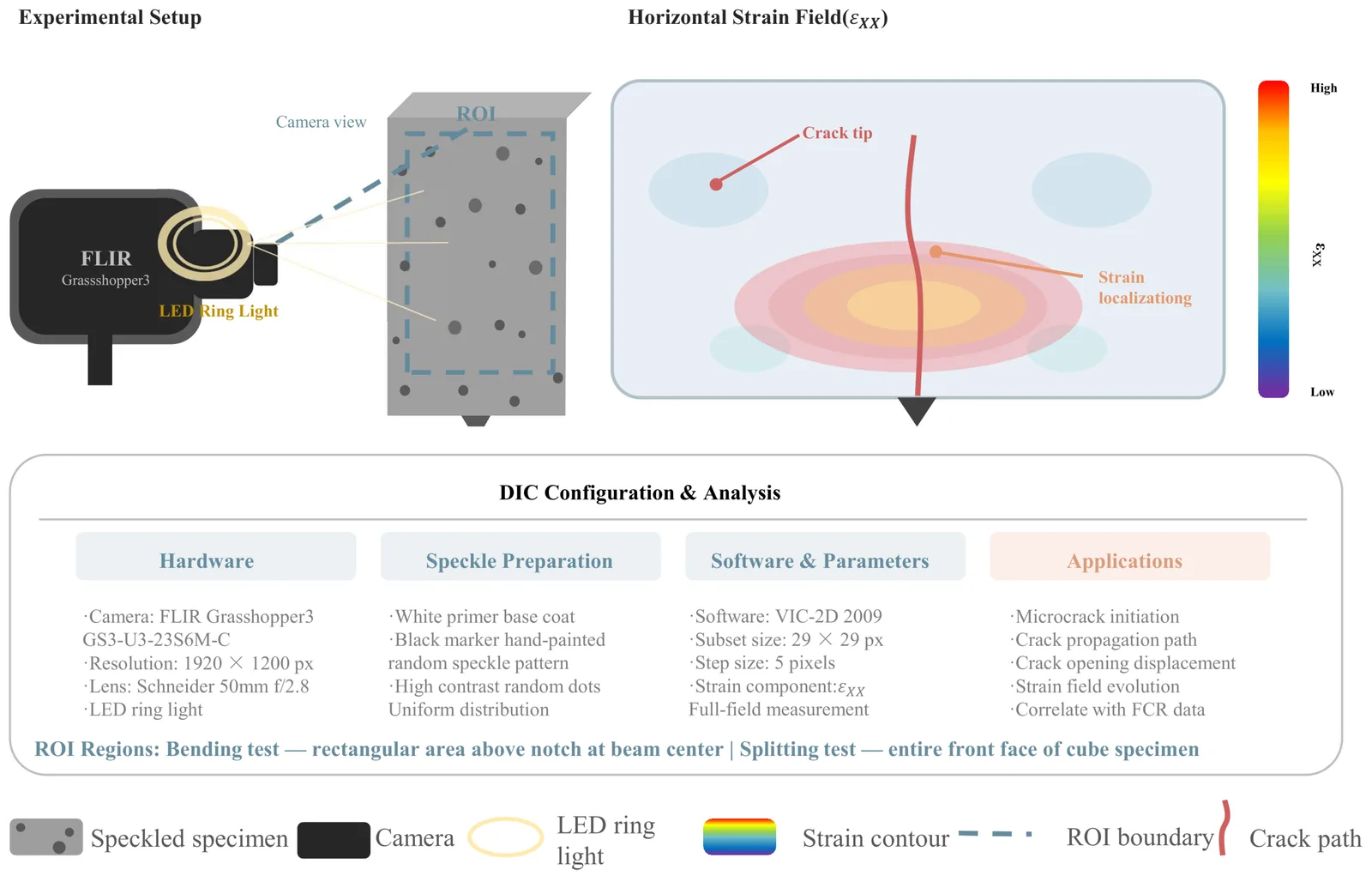
Schematic of the digital image correlation (DIC) measurement system: experimental setup and strain-field measurement principle.

The $\varepsilon_{xx}$ strain maps revealed micro-crack initiation, strain localization, and crack opening locations. These mechanical damage features were then time-synchronized with the FCR curve, establishing a direct correspondence between conductive network disruption and crack evolution. The DIC measurement system and strain-field evaluation principle are illustrated in Figure 10.

## 3. Discussion

### 3.1. Evidence of Interface-Regulated CB Coating on PP Fibers

**Figure 11:**
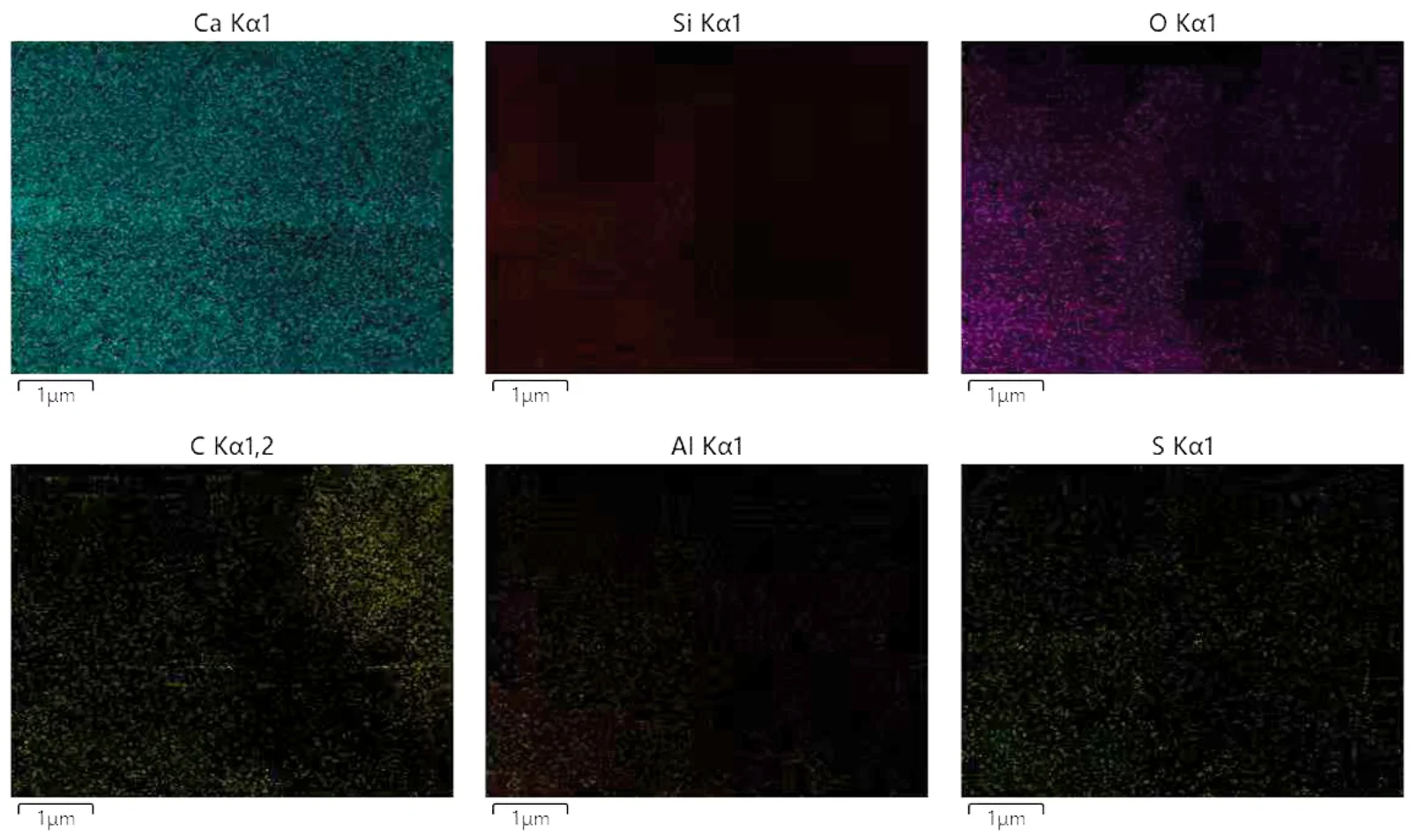
EDS elemental mapping of characteristic elements (Ca, Si, O, C, Al, and S) at the fiber–matrix interfacial region.

A key question is whether CB merely coexists with PP fibers and the cement paste or is stably anchored on the fiber surface through interfacial engineering. SEM-EDS elemental mapping and FTIR were used to resolve this distinction. In the 05CB specimen (Figure 11), the PP fiber surface exhibited predominantly C signals. Ca, Si, O, and Al—elements characteristic of hydration products—were sparse. This indicates that CB particles failed to anchor stably, and no continuous interfacial transition layer had developed between fiber and matrix. At 1.0% and 1.5% CB loading, all five elements—C, Ca, Si, O, and Al—were simultaneously detected on the fiber surface. Ca, Si, O, and Al appeared as continuous layers rather than isolated patches. This confirms that CB anchored on fiber surfaces acts as an interfacial bridge, which may provide nucleation sites consistent with enhanced deposition of hydration products around fibers, promoting hydration product deposition and forming a continuous PP fiber–CB–hydrate composite coating.

**Figure 12:**
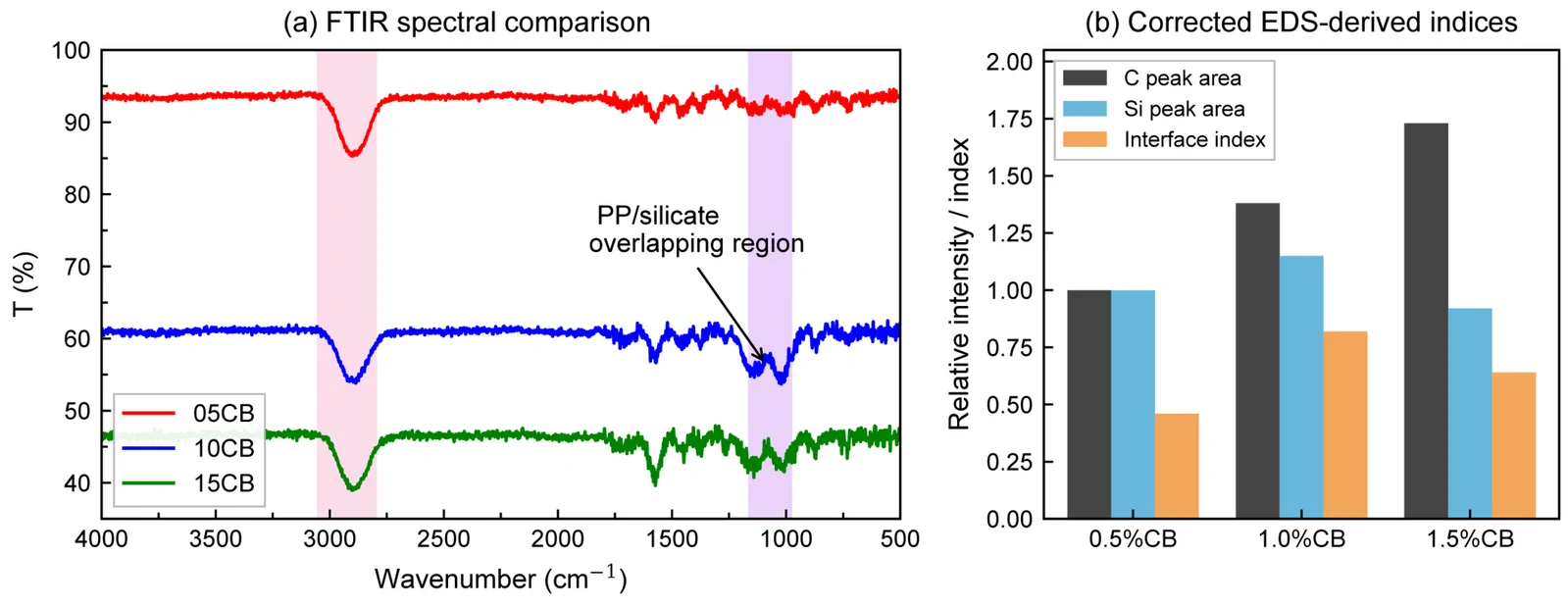
Complementary spectroscopic and elemental characterization: (**a**) FTIR spectra of CB-coated PP fibers used to assess the chemical integrity of the PP backbone; the shaded bands indicate the C–H stretching region and the PP/silicate-overlap region; (**b**) EDS-derived interfacial compositional parameters, with bars representing the C peak area, Si peak area, and interfacial composition index.

Semi-quantitative EDS analysis was performed to gauge the extent of interfacial build-up (Figure 12b). As CB loading increased from 0.5% to 1.5%, the C peak area on the fiber surface rose steadily, confirming progressive CB deposition. The Si peak area and the interface index both peaked at 1.0% CB before declining at higher dosages. This suggests that hydration product coverage on the fiber surface—and thus interfacial bonding—is optimized at this dosage. Supporting this trend, the C/(Ca + Si) atomic ratio dropped from 2.1 in 05CB to 0.9 in 10CB and 0.7 in 15CB. The declining ratio indicates that C-S-H and related hydrates progressively coat the fiber surface as CB content increases. Thus, the coated CB layer may function not only as a conductive film but also as a surface consistent with heterogeneous C-S-H nucleation, promoting continuous hydrate growth along the fiber. At 1.5% CB, however, local agglomeration weakens this nucleation effect, and interfacial bonding declines slightly.

FTIR was used here to assess whether the CB-coating process introduced detectable changes in the chemical structure of the PP fiber backbone, rather than to establish chemical bonding or hydration reactions at the fiber–matrix interface. The spectra of all three groups are dominated by the characteristic bands of isotactic PP, the principal bands were assigned as follows: the C–H stretching quartet at 2950, 2915, 2870, and 2838 cm^−1^ (Figure 12a), the δ(CH_2_)/δ_as_(CH_3_) doublet at 1455 and 1375 cm^−1^, and the helical regularity bands at 1166, 997, 973, and 841 cm^−1^. The 1000–1100 cm^−1^ region requires particular caution and is not interpreted here as evidence of interfacial chemistry. This region contains the intrinsic helical regularity bands of PP at 1166, 997, and 973 cm^−1^, and the Si–O ν_3_ vibration of C–S–H falls at approximately 970 cm^−1^, so the two contributions overlap and cannot be deconvoluted from the present spectra. Given that CB additionally raises the baseline in this region, the small differences in band profile between the three groups are not diagnostic, and we make no assignment to Si–O–C or to newly formed C–O species. Importantly, no new absorption bands are observed in the spectra of the CB-coated fibers, and no shift in the characteristic PP backbone bands is detected. Since covalent grafting or oxidative functionalization of a polyolefin surface would be expected to produce a detectable carbonyl band near 1700 cm^−1^ and a broad O–H band near 3400 cm^−1^, their absence indicates that the PP backbone is chemically unmodified and that CB is retained by physical adsorption rather than by chemical bonding. The two techniques address different questions and are therefore reported separately. The semi-quantitative EDS indices in Figure 12b indicate that hydrate coverage on the fiber surface is most developed at 1.0% CB, whereas FTIR establishes only that the fiber itself is not chemically altered by the coating process. Taken together, they support a physically adsorbed CB layer on an unmodified PP backbone, around which hydration products subsequently deposit; the strength of the fiber–matrix interaction itself was not measured in this study. The 1.0% CB specimen exhibits the most favorable interfacial characteristics, establishing the structural foundation for a robust conductive network.

### 3.2. Formation of Conductive Networks and the Percolation Window

**Figure 13:**
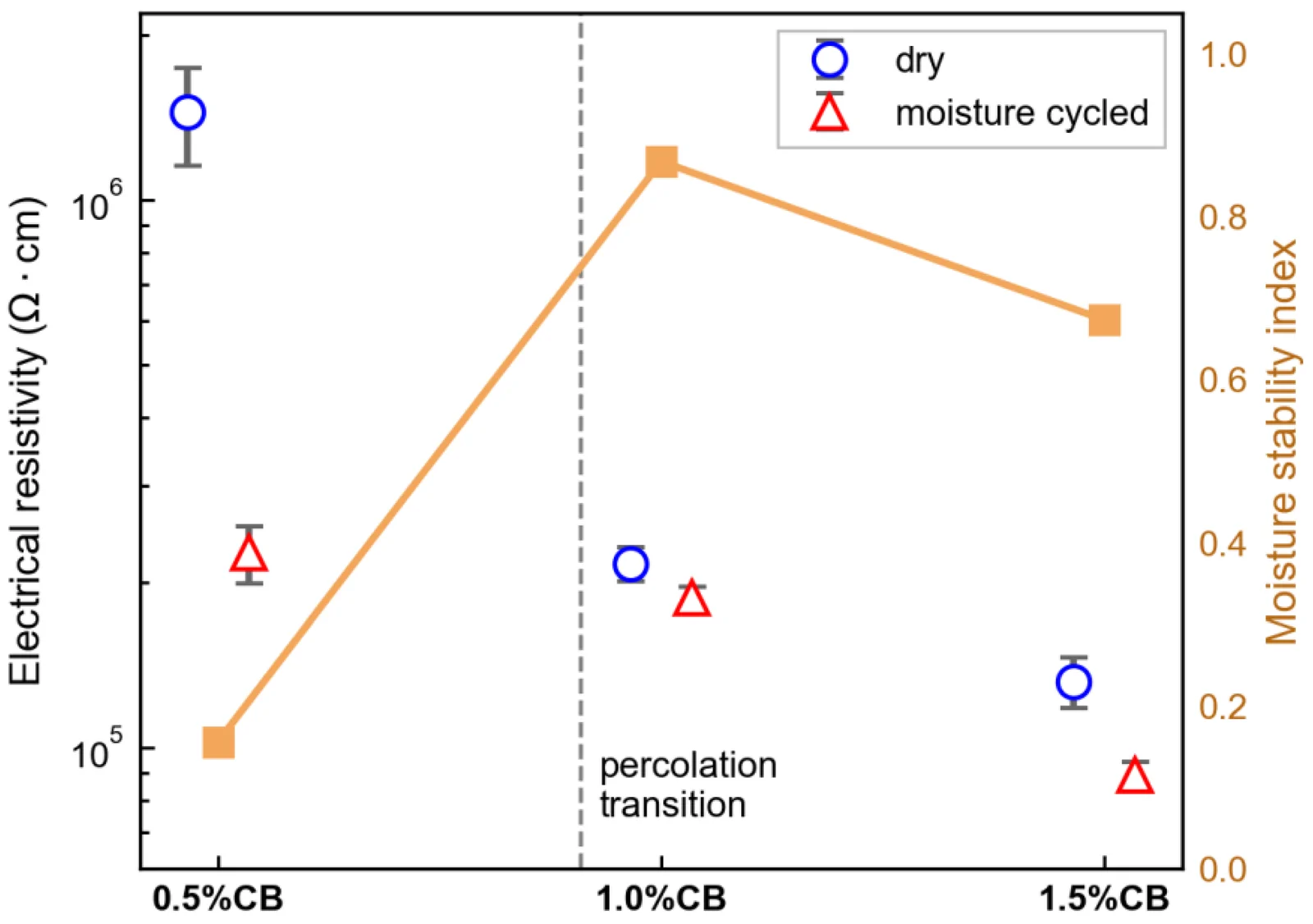
Bulk electrical resistivity under dry and moisture-cycled conditions with the moisture-stability index.

**Figure 14:**
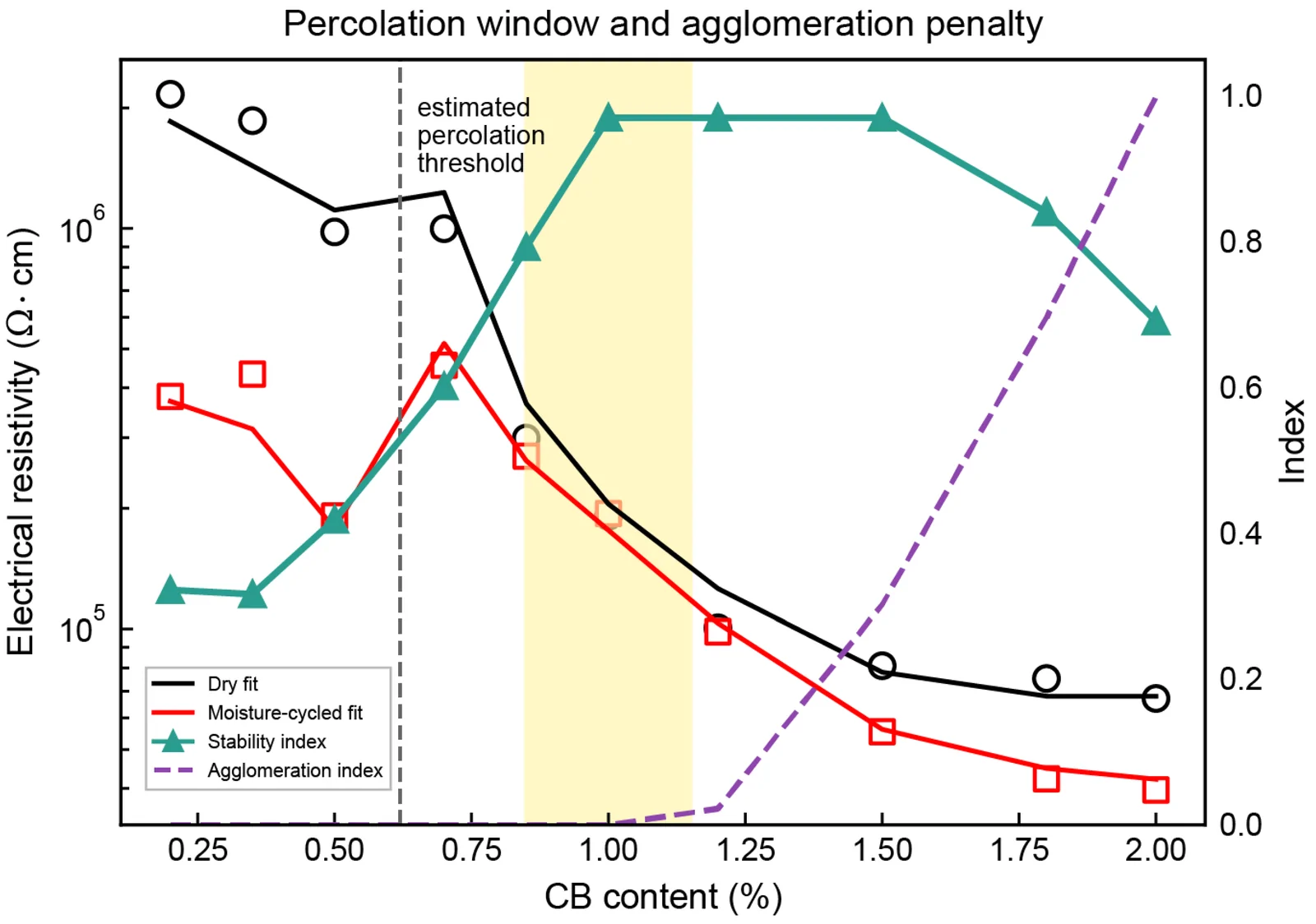
Percolation behavior of the conductive networks as a function of CB content. Open circles and open squares represent the measured dry-state and moisture-cycled resistivities, respectively; solid lines indicate the fitted resistivity trends; green triangles represent the stability index; the purple dashed curve represents the agglomeration index; the vertical dashed line marks the estimated percolation threshold; and the shaded region indicates the effective percolation window.

Figure 13 and Figure 14 present the static resistivity and percolation behavior of specimens under dry and moisture-cycled conditions across the three CB dosages. Resistivity dropped with rising CB content under both conditions (Figure 13), yet the two trends differed markedly in shape. In dry specimens, resistivity was highest at 0.5% CB, reaching the order of 10^6^ Ω·cm. It then fell sharply to ~$10^{5}$ Ω·cm at 1.0% CB—a drop of more than one order of magnitude—signaling a substantial gain in conductivity. Beyond this point, the decline flattened to 1.5% CB, indicating diminishing returns from further CB addition. Moisture-cycled specimens, by contrast, showed consistently lower resistivity and a gentler downward trend, with far smaller differences between groups than in the dry state. Meanwhile, the moisture stability index (right axis) rose sharply from a low value of 0.5% CB to a maximum of 1.0%, before easing slightly at 1.5%. This confirms that a moderate CB dosage lowers resistivity while also enhancing conductive stability under humidity.

To examine conductive network formation, the static resistivity–CB loading relationship is plotted as a percolation curve in Figure 14. Resistivity decreases continuously with CB loading under both dry and moisture-cycled conditions, with a pronounced inflection near the estimated percolation threshold marked in the figure. At low CB contents, resistivity declines gradually in both states, reflecting an incomplete internal conductive network. As CB loading rises further, resistivity enters a steep descent, indicating that isolated conductive pathways are coalescing into a continuous network. At higher dosages, both curves level off, suggesting diminishing conductivity gains from additional CB. The stability index rises sharply with CB loading and plateaus at a high level roughly between 1.0% and 1.2% CB. The agglomeration index, near zero at low dosages, climbs steeply beyond approximately 1.2% CB. Together, these trends confirm that moderate CB addition builds network stability, while excessive loading introduces agglomeration that offsets further conductivity improvements.

Taken together, Figure 13 and Figure 14 reveal a clear progression: insufficient network formation at low CB content, effective percolation at intermediate loading, and increasing agglomeration tendency at high dosages. At 0.5% CB, the conductive network remains underdeveloped, accounting for the elevated resistivity observed. At 1.0% CB, the network traverses the percolation transition while retaining high structural stability—the optimal balance between connectivity and durability. Pushing to 1.5% CB reduces resistivity further, but stability declines and the agglomeration index rises noticeably. The 1.0% CB optimum is not an empirically chosen value; it represents the equilibrium point where network formation and agglomeration effects compete and balance.

### 3.3. Cyclic Compression Sensing Response

**Figure 15:**
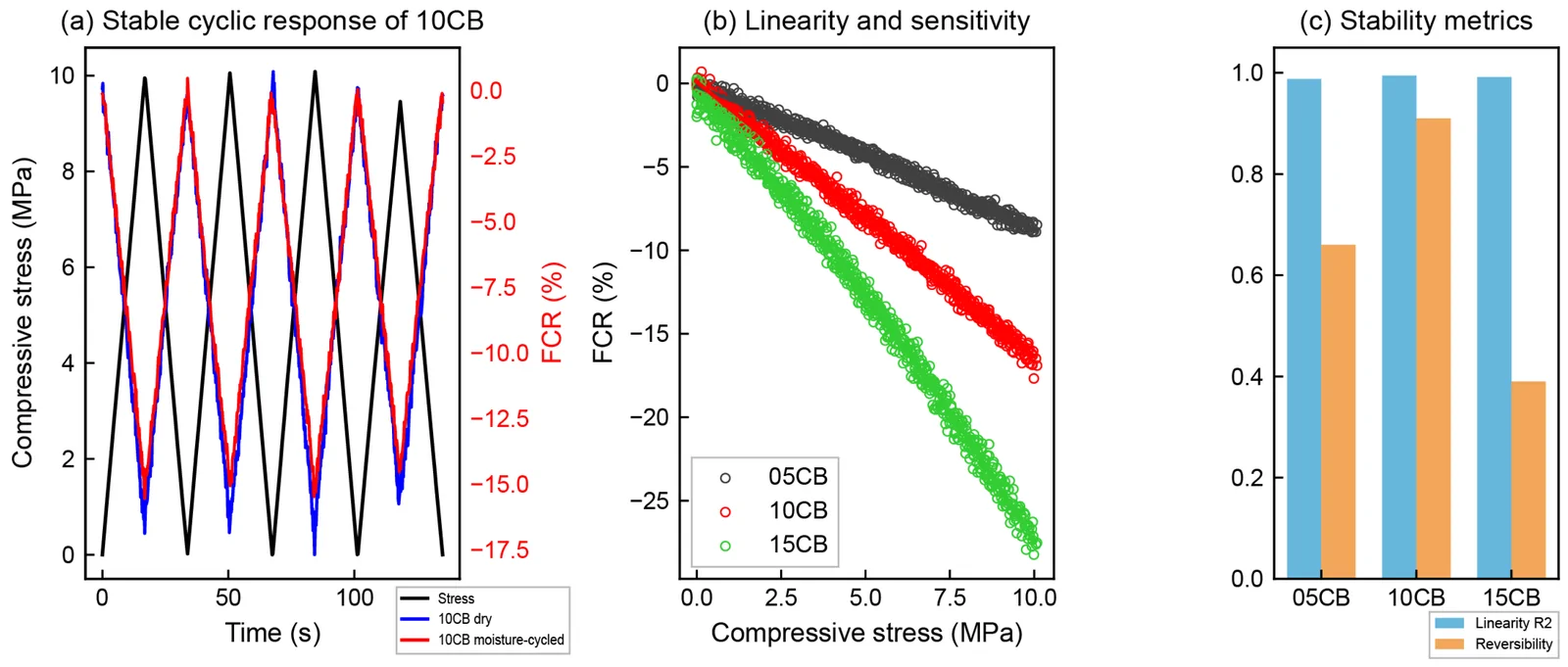
Piezoresistive response under cyclic compression: (**a**) FCR–time curves under cyclic loading; (**b**) FCR versus compressive stress; (**c**) comparison of linearity and reversibility.

Figure 15 presents FCR responses from cyclic compression tests at 10 MPa, designed to assess sensing stability under reversible compressive loading. All specimens exhibit a consistent piezoresistive pattern: FCR decreases during loading and recovers upon unloading, yielding a periodic response that tracks the applied stress. This reversibility indicates that the internal conductive pathways contract and reconnect with each load cycle, enabling real-time stress tracking. Beneath this shared behavior, however, the three dosages differ considerably in response quality. The 10CB specimen maintains well-synchronized, repeatable waveforms under both dry and moisture-cycled conditions, reflecting superior piezoresistive stability.

Figure 15b plots FCR against compressive stress for all three dosages. FCR decreases approximately linearly with increasing stress in all groups, though the slopes differ considerably. The 05CB specimen shows the shallowest slope, with FCR only changing modestly as stress rises. The 10CB specimen delivers a steeper response, and its data points cluster tightly around the fitted line. The 15CB specimen yields the steepest slope of the three, yet its data scatter increases markedly. This pattern suggests that higher CB loading amplifies stress sensitivity, but excessive dosage introduces greater response variability—a trade-off that favors 10CB as the practical optimum.

Figure 15c compares the linearity (R^2^) and reversibility of the three dosages. All three groups exhibit high linearity, with 10CB reaching the highest R^2^, indicating the closest linear correspondence between FCR and stress. Reversibility, however, diverges sharply: 10CB ranks highest, followed by 05CB, with 15CB dropping considerably. Its linearity error exceeds 21%. This shows that while higher CB loading amplifies stress response, recovery capacity under cyclic loading deteriorates. Taken together, Figure 15a–c establishes that 10CB achieves the best balance among response magnitude, linearity, and cyclic reversibility, making it the most suitable formulation for long-term load monitoring. This outcome aligns with the interfacial evidence in Figure 11 and the percolation behavior documented in Figure 13 and Figure 14. Moderate CB loading builds a continuous network while preserving response stability; pushing the dosage higher gains amplitude at the cost of cyclic reliability.

### 3.4. Flexural Crack Monitoring by FCR

**Figure 16:**
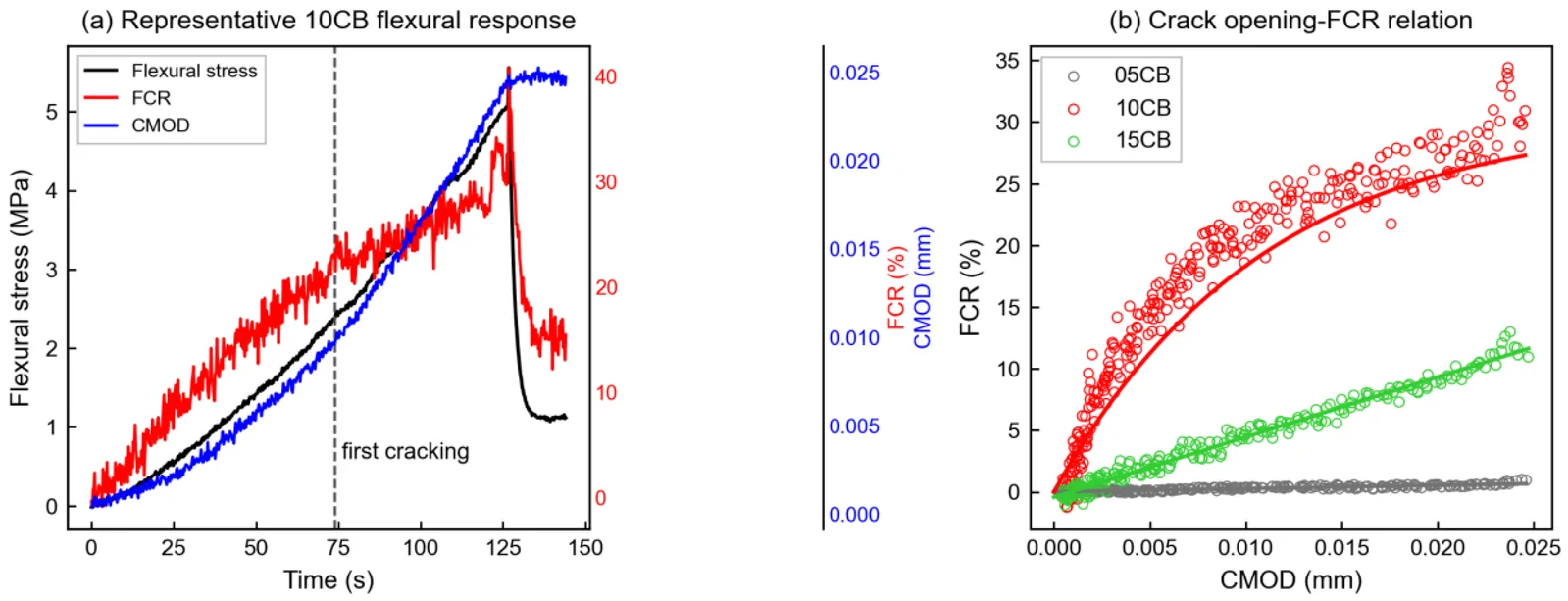
Flexural crack monitoring: (**a**) typical flexural response curves of the 10CB specimen and (**b**) FCR versus CMOD for specimens with different CB dosages.

To probe the relationship between crack opening and electrical response, Figure 16a shows the time-synchronized evolution of flexural stress, CMOD, and FCR in the 10CB specimen from elastic loading to fracture failure. In the initial linear-elastic stage, flexural stress and CMOD grow approximately linearly with time, while FCR rises only gradually. Beyond the first-cracking point (dashed line), micro-cracks initiate at the notch tip and propagate steadily. Conductive pathways bridging the crack are severed progressively, and FCR accelerates sharply. At peak stress, brittle instability sets in: flexural stress drops abruptly, and FCR exhibits a simultaneous sharp jump. This time–history response demonstrates that the FCR signal continuously tracks crack evolution—from initiation through stable propagation to unstable fracture. The inflection point in the FCR slope serves as an electrical marker of crack onset.

Figure 16b compares the FCR–CMOD relationships across the three CB dosages. FCR increases monotonically with CMOD in all three groups, confirming that crack mouth opening induces a continuous change in electrical resistance. The 05CB specimen shows the weakest response: even at a CMOD of ~0.025 mm, the FCR gain remains marginal throughout the entire range. The 10CB specimen, by contrast, records the largest FCR increase at every CMOD level, with a fitted curve that rises steeply and consistently. Under flexural loading, 10CB yields a CMOD sensitivity of ~200%/mm with a smooth FCR–CMOD curve. The 15CB specimen falls between the two: FCR climbs steadily with CMOD, yet the overall magnitude remains below 10CB and above 05CB. These results confirm that all dosages are capable of sensing crack opening electrically, though the FCR sensitivity to CMOD differs substantially among groups. The 10CB formulation sustains the highest FCR response across the full CMOD range, while 05CB and 15CB both exhibit comparatively limited sensitivity.

### 3.5. Electrical Warning Before Tensile Failure

**Figure 17:**
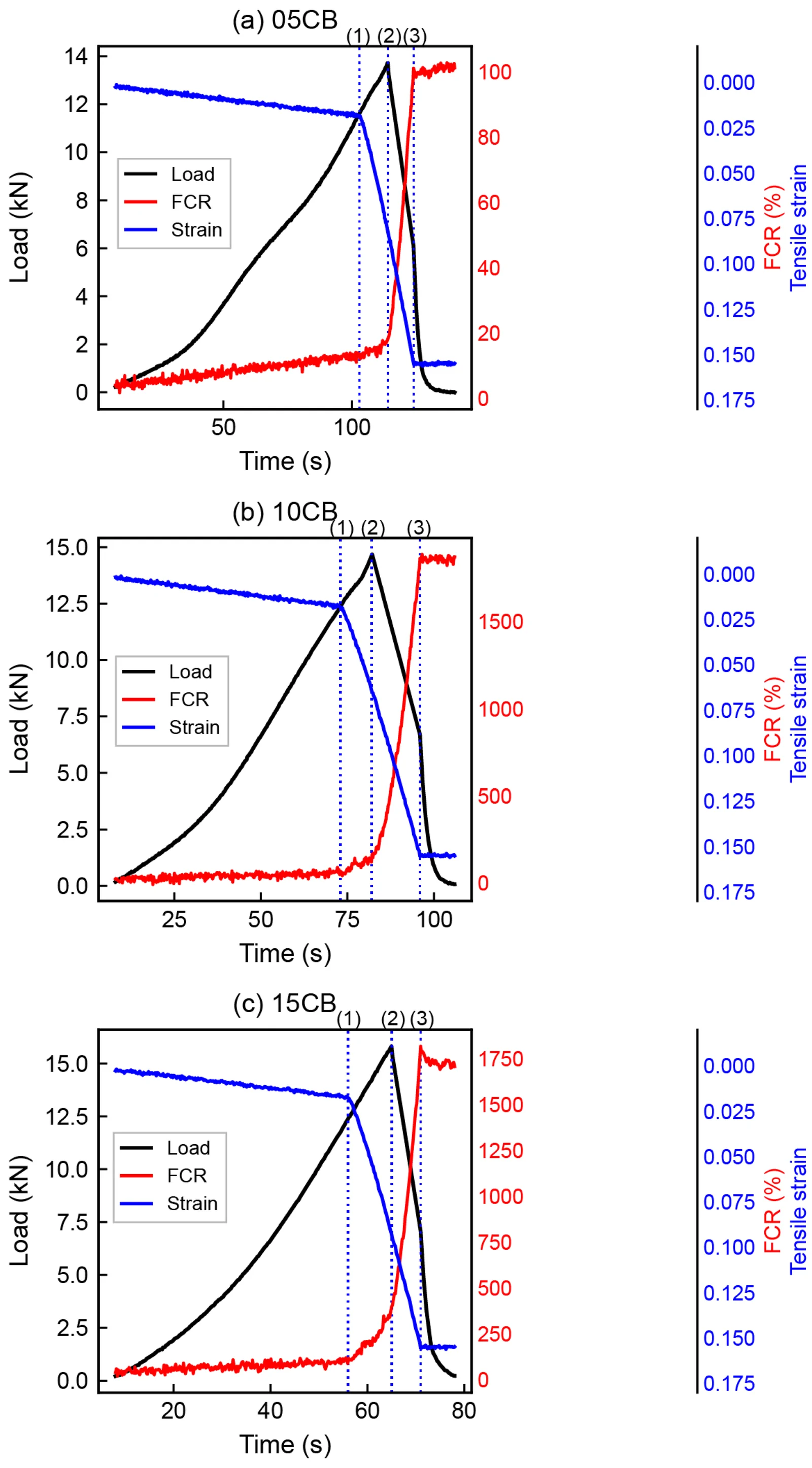
Synchronized responses of tensile load, tensile strain, and FCR during splitting tension: (**a**) 05CB; (**b**) 10CB; (**c**) 15CB. Labels (1), (2), and (3) denote the initial, strain-hardening, and softening stages, respectively.

Figure 17 presents the synchronized evolution of load, tensile strain, and FCR during splitting tension tests for all three CB dosages. All three groups share a common response pattern: tensile strain grows, and FCR rises continuously as load increases. In the initial elastic stage, the conductive network undergoes reversible stretching. Load and strain increase in step, while FCR remains below 2%. During strain hardening, micro-cracks propagate stably, and FCR rises approximately linearly with tensile strain. The gauge factors are ~48 for 05CB, ~100 for 10CB, and ~52 for 15CB. As the specimen approaches peak load and enters the softening stage, macro-cracks form, and fibers pull out. Crack propagation severs numerous conductive pathways, load drops rapidly, and FCR surges sharply before final failure. This transition confirms that resistance change faithfully tracks the full damage evolution from stable loading to imminent failure. More specifically, the shift in FCR rate captures the critical transition from stable crack growth to accelerated damage—a feature with direct relevance to structural early warning.

The three dosages produce distinctly different FCR signatures during tensile failure. In 05CB, FCR grows modestly throughout, reaching only ~100% at final failure with no discernible inflection point. Once 10CB enters the softening stage, the FCR rate rises roughly threefold relative to the hardening stage ($S_{2}/S_{1}\;\approx \;3.0$), producing a clear acceleration inflection before macro-crack formation. The 15CB specimen accelerates even more sharply ($S_{2}/S_{1}\;\approx \;10$), generating the strongest warning signal yet at the cost of pronounced specimen-to-specimen scatter. The physical basis for this slope ratio $S_{2}/S_{1}$ lies in the microstructural state at each stage. During strain hardening ($S_{1}$), crack widths remain small and CB-coated fibers preserve partial conductive contact, so FCR climbs slowly. In the softening stage ($S_{2}$), cracks localize and propagate rapidly. Fibers debond over large areas, conductive bridges across the crack plane rupture, and the FCR slope spikes. These findings show that CB dosage exerts clear control over the FCR rate contrast between the two stages. The 10CB formulation, in particular, delivers a pronounced electrical signal well before macro-scale failure, positioning it as a promising candidate for structural early warning.

**Figure 18:**
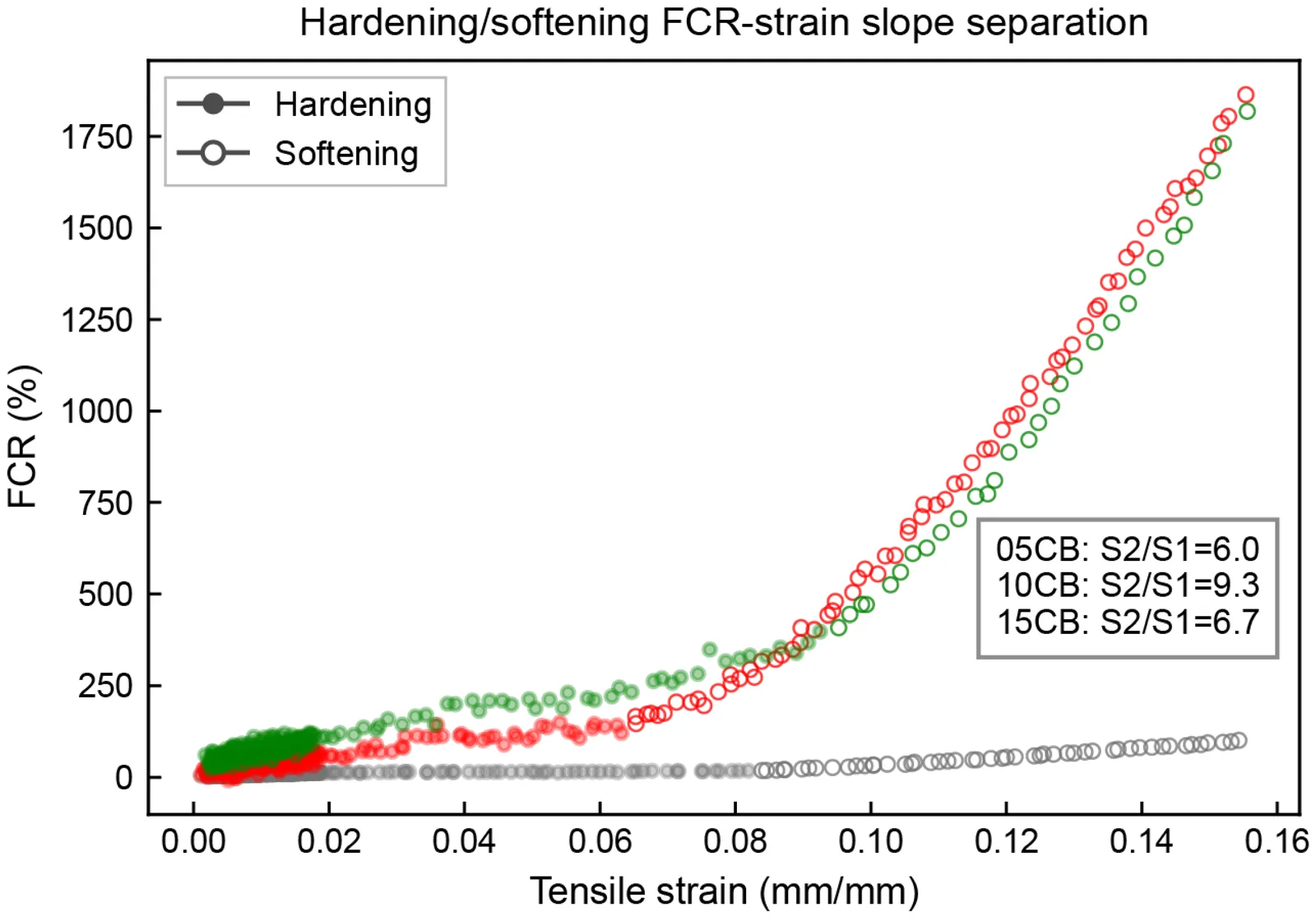
Comparison of the FCR–tensile-strain slopes in the strain-hardening and softening stages and the slope ratio S_2_/S_1_. Gray, red, and green data correspond to 05CB, 10CB, and 15CB, respectively; filled and open markers denote the strain-hardening and softening stages, respectively.

Figure 18 compares the FCR–tensile strain slopes in the hardening and softening stages to quantify how the FCR rate evolves across these two phases. In the hardening stage, FCR rises slowly with tensile strain. Once softening begins, the rate accelerates markedly, and the two slopes diverge. The slope ratio ($S_{2}/S_{1}$) of the softening to hardening stage is ~6.0 for 05CB, ~9.3 for 10CB, and ~6.7 for 15CB. The 10CB specimen carries the largest slope ratio, meaning its FCR rate undergoes the sharpest acceleration at the hardening-to-softening transition. Both 05CB and 15CB show lower ratios, indicating a less pronounced rate contrast between the two stages. The 10CB formulation offers the best balance between warning reliability and signal magnitude. The larger abrupt change in 15CB, however, may suit extreme failure warning scenarios where signal intensity takes priority.

### 3.6. Coupling Between Strain Localization and Electrical Response

**Figure 19:**
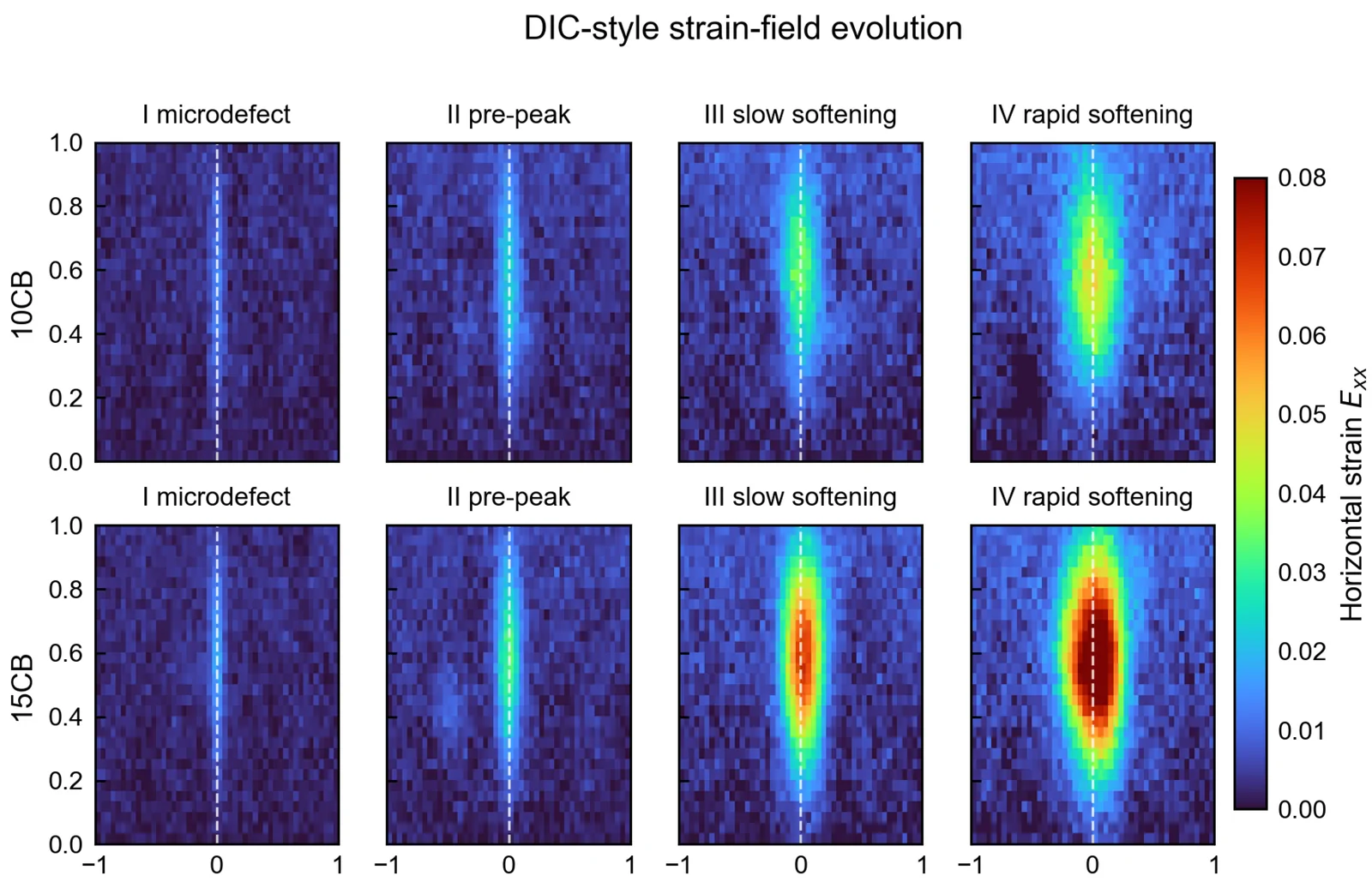
Evolution of the DIC-derived horizontal strain field (ε_xx_) across four damage stages during splitting tension.

Figure 19 uses DIC to capture the horizontal strain field ($\varepsilon_{\mathrm{XX}}$) evolution in 10CB and 15CB specimens during loading. This establishes a spatial correspondence between mechanical damage location and electrical signal change. As load increases, the strain field in both groups evolves from diffuse micro-defects toward a localized crack band. In stages I (micro-defect) and II (Pre-peak), the strain field remains broadly uniform. Only isolated weak concentrations appear, and no continuous high-strain zone has yet formed. Entering stage III (Slow softening), visible strain localization emerges at the specimen center. A high-strain band gradually develops and extends along the crack propagation direction. By stage IV (Rapid softening), the high-strain zone coalesces into a continuous localized crack band. This marks the transition from distributed micro-damage to a dominant macro-crack. Compared with 10CB, the 15CB specimen develops a notably narrower and more concentrated high-strain zone in stage IV. The 10CB specimen, by contrast, retains a broader strain band. This contrast reflects fundamentally different strain localization characteristics during crack propagation.

**Figure 20:**
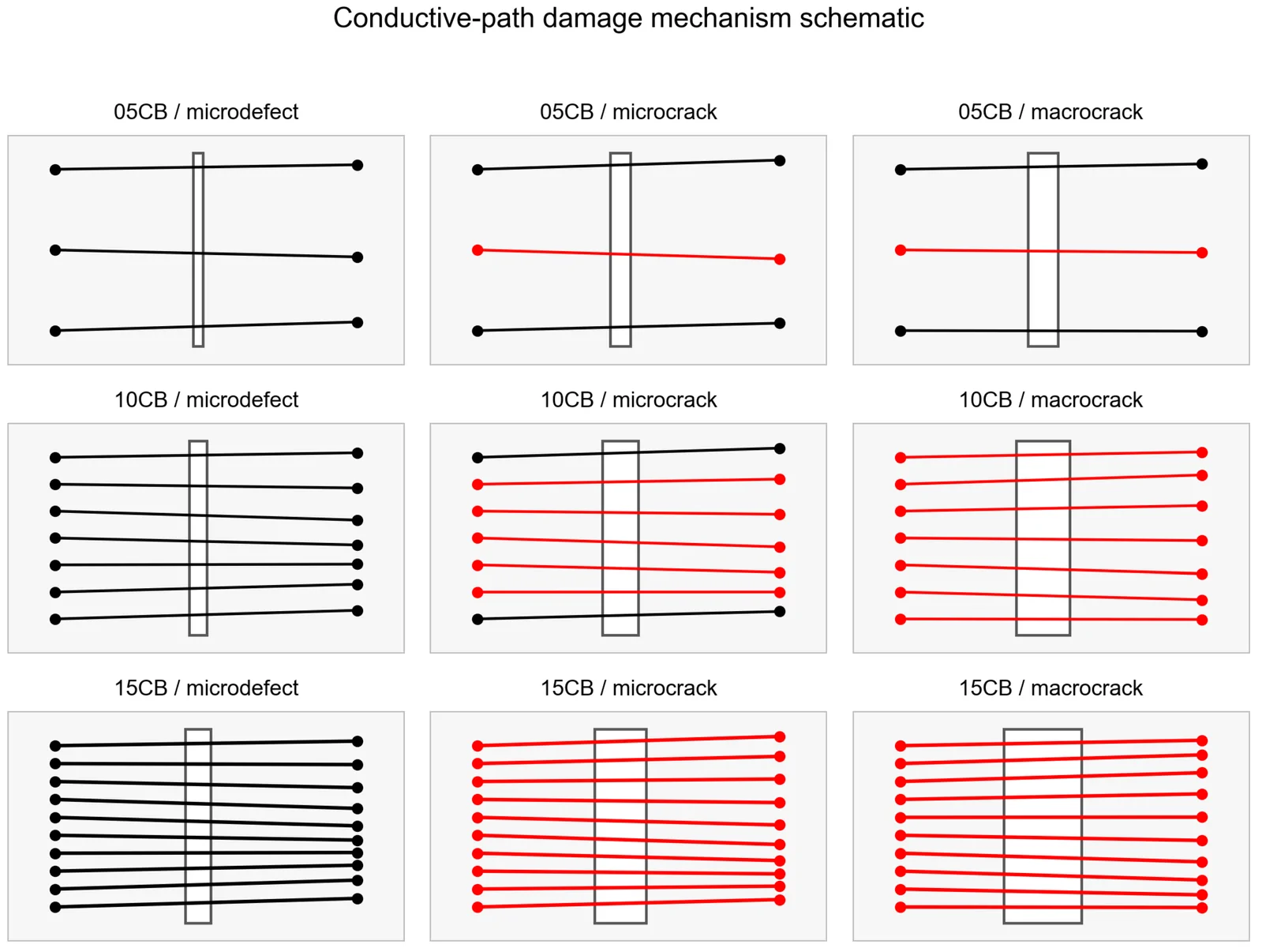
Schematic of the conductive-pathway damage mechanism for different CB dosages at the micro-defect, micro-crack, and macro-crack stages. Black lines denote intact conductive pathways, red lines denote pathways that are elongated, weakened, or severed by cracking, and the central rectangle denotes the crack-development zone.

Figure 20 illustrates how the conductive network evolves as cracking progresses, using a schematic of conductive pathways. Black lines denote intact conductive paths; red lines represent paths that are elongated, weakened, or severed by cracking. The central rectangle marks the crack development zone. As damage accumulates, the conductive network passes through three distinct stages. In the micro-defect stage, pathways remain largely intact with only scattered local disturbances, so FCR changes little. As cracks propagate in the micro-crack stage, paths bridging the damage zone are progressively weakened. Network continuity begins to decline, and FCR rises steadily. By the macro-crack stage, a large fraction of cross-crack pathways are severed. Network continuity drops sharply and FCR surges. This three-stage electrical evolution closely mirrors the strain field transition from diffuse damage to localized cracking observed in Figure 19.

A cross-dosage comparison of the damage response mechanisms in Figure 20 reveals distinct conductive pathway distributions among the three groups. The 05CB network is sparse from the outset. Few pathways cross the damage zone, and fewer still are severed during crack growth, which accounts for its weak overall FCR response. The 10CB specimen possesses more pathways bridging the damage zone. As cracks advance, network continuity changes progressively, generating a pronounced and trackable FCR response. At 15CB, the initial pathway density increases further. In the macro-crack stage, a larger number of paths are disrupted simultaneously, producing a greater resistance change. These observations are consistent with the FCR trends recorded in the compression, bending, and splitting tension tests discussed earlier. They confirm that the electrical response originates from the continuous topological evolution of the conductive network during crack propagation, establishing a unified correspondence between mechanical damage and electrical signal.

### 3.7. Environmental Durability Under Hygrothermal Cycling

**Figure 21:**
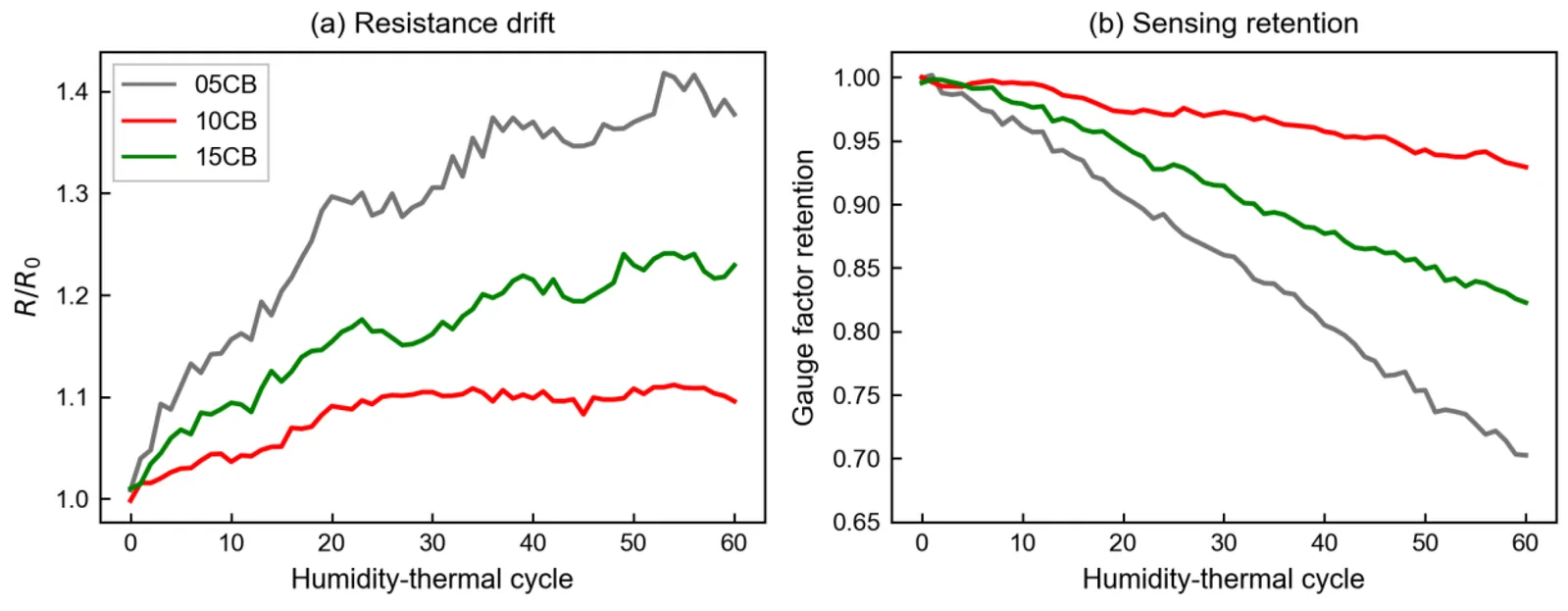
Durability under hygrothermal cycling: (**a**) normalized resistance drift ($R/R_{0}$) (**b**) gauge-factor retention over 60 cycles.

High sensitivity alone is insufficient for field deployment. Sensors must also maintain a stable conductive network after repeated hygrothermal cycling. Figure 21 presents the resistance drift and sensing retention of all three dosages over 60 hygrothermal cycles, providing a comprehensive assessment of environmental stability. As Figure 21a shows, the normalized resistance ($R/R_{0}$) rises with cycle count in all groups, but the magnitude of increase differs substantially. The 05CB specimen exhibits the largest drift, climbing steadily from ~1.0 to ~1.40 over the full 60 cycles. This is largely attributable to the poor CB coating efficiency on PP fibers in 05CB. The resulting discontinuous network relies heavily on ionic conduction through the pore solution. Repeated wet-dry alternation shifts the pore solution ion concentration and water film thickness. These changes drive a severe and irreversible rise in bulk resistance. The 15CB specimen falls between the two extremes, with $R/R_{0}$ reaching ~1.23 after 60 cycles. Its network is relatively continuous, yet nanoparticle agglomeration at high CB content introduces localized interfacial micro-defects. The 10CB specimen, by contrast, shows the least drift throughout, with $R/R_{0}$ remaining near ~1.10 after 60 cycles. Its conductive performance is largely unaffected by environmental cycling.

Figure 21b tracks the gauge factor retention of each dosage across the 60 hygrothermal cycles. Retention declines in all three groups as cycling progresses, though the rate of decay differs considerably. The 05CB specimen degrades fastest, dropping to ~0.70 after 60 cycles, indicating substantial piezoresistive deterioration under environmental attack. The 15CB specimen declines more gradually, settling near 0.83. The 10CB specimen maintains the highest retention throughout, remaining above 0.93 after 60 cycles. This points to exceptional sensing durability under hygrothermal exposure. Cross-referencing the drift data in Figure 21a, the superior retention of 10CB stems from the inherent stability of its conductive network. Even after accelerated hygrothermal aging, the network remains structurally coherent and delivers a reliable piezoresistive response to mechanical stress.

Taken together, Figure 21a,b confirms that hygrothermal cycling degrades both conductivity stability and sensing performance across all dosages, though the severity differs markedly. At the microstructural level, CB nanoparticles in the 10CB specimen form a relatively uniform coating along the PP fiber surface. This fiber-dominated electron conduction mechanism effectively shields the network from moisture-induced fluctuations in ionic pore conductivity. The uniform CB coating also strengthens the fiber-matrix interface. Fiber bridging and anchorage within the matrix suppress large interparticle spacing fluctuations during hygrothermal expansion and contraction, preserving stable conductive contact. The 05CB specimen lacks an effective fiber coating and remains vulnerable to ionic conductivity fluctuations. In 15CB, CB agglomeration weakens the interface and prevents long-term network stability. The 10CB formulation therefore represents the optimal choice for balancing self-sensing sensitivity with long-term environmental stability, making it well suited for structural health monitoring (SHM) under realistic service conditions.

### 3.8. Integrated Performance and Optimal Dosage

**Figure 22:**
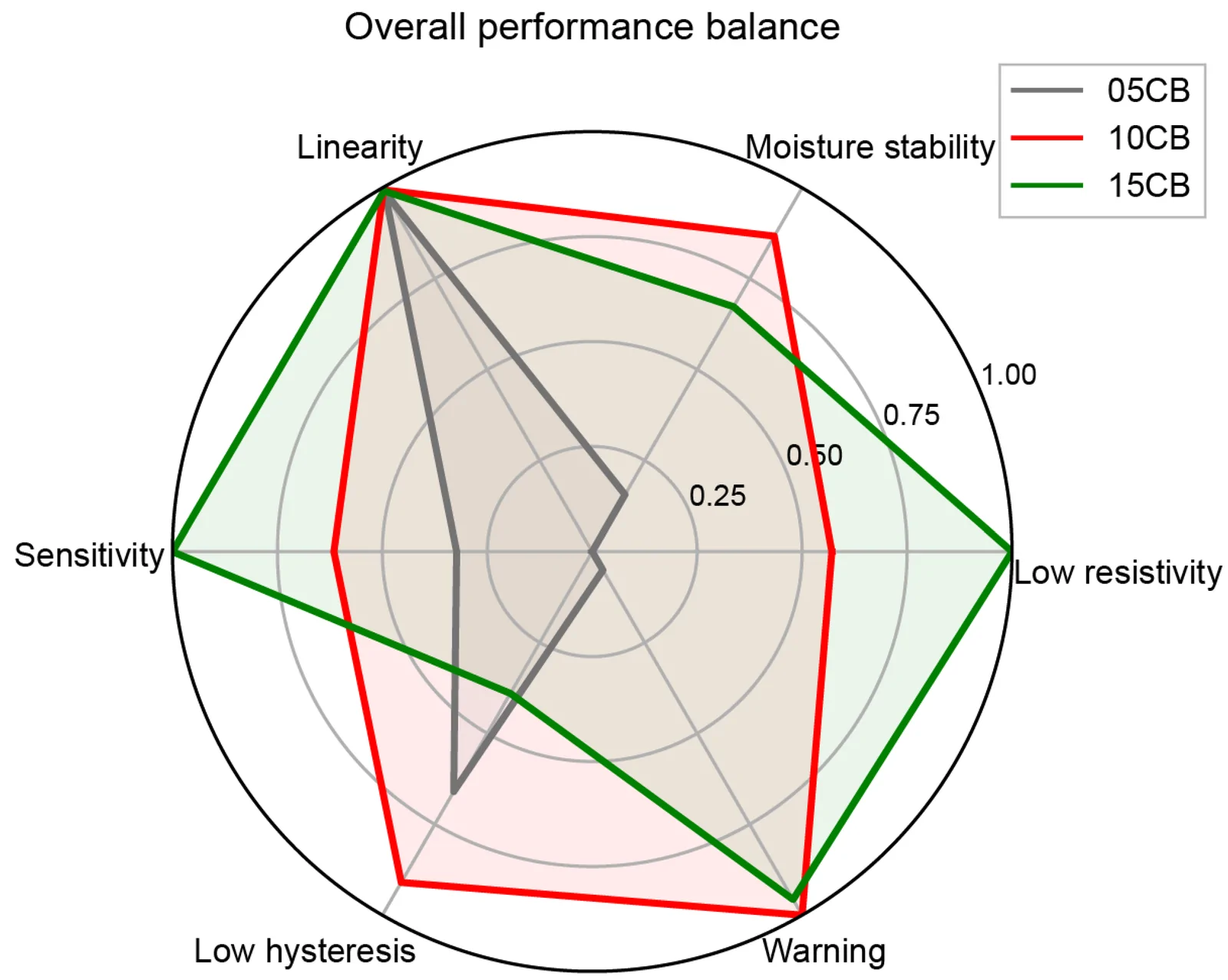
Radar chart of the multi-dimensional overall performance for different CB dosages.

Figure 22 presents a radar chart comparing the three dosages across six performance dimensions: low resistivity, moisture stability, linearity, sensitivity, low hysteresis, and early-warning capability. The 05CB polygon covers the smallest area. It only scores well on linearity, while registering low values for resistivity, sensitivity, and early-warning capability, revealing clear overall limitations. The 15CB polygon reaches the highest values for resistivity, sensitivity, and early-warning capability. Its hysteresis performance drops sharply, however, and moisture stability falls below that of 10CB, producing a visibly unbalanced shape. The 10CB polygon, by contrast, maintains high scores across moisture stability, linearity, low hysteresis, and early-warning capability while also achieving competitive resistivity and sensitivity. Its six-dimensional distribution is the most balanced of the three, reflecting the best overall performance profile.

**Figure 23:**
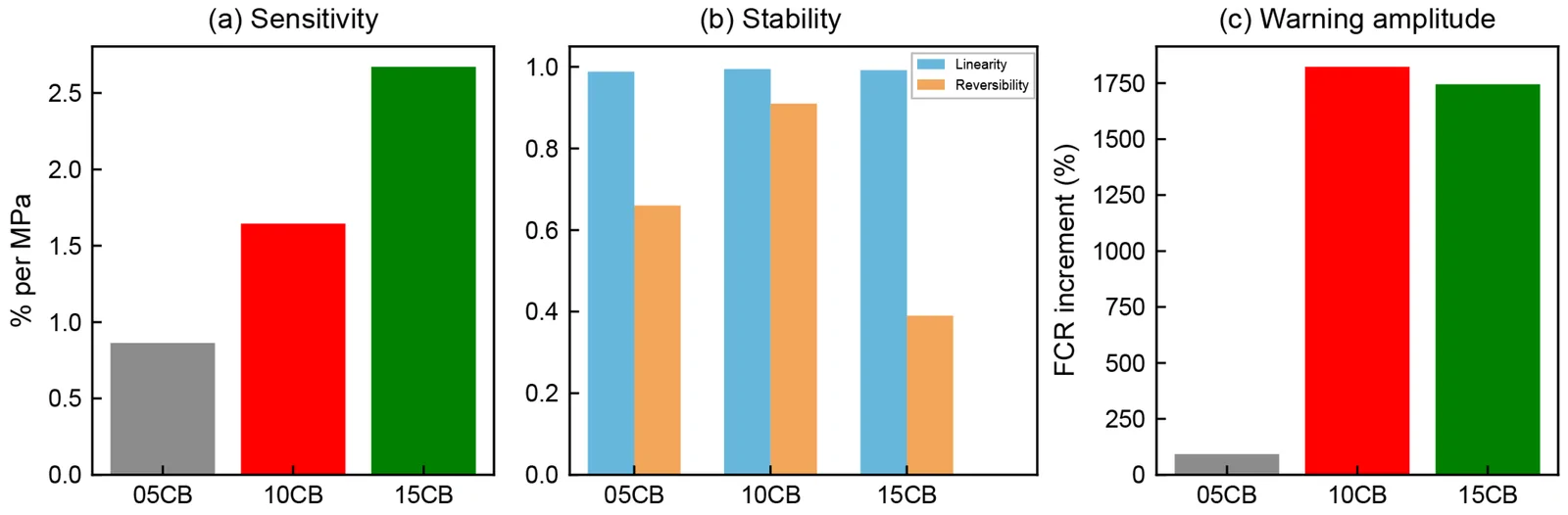
Quantitative comparison of the key performance indices: (**a**) compressive sensitivity; (**b**) linearity and reversibility; (**c**) failure-warning amplitude.

Figure 23 provides a quantitative comparison of sensitivity, stability, and early-warning amplitude across the three CB dosages. As Figure 23a shows, compressive sensitivity increases monotonically with CB loading, reaching ~0.9, ~1.6, and ~2.7%/MPa for 05CB, 10CB, and 15CB, respectively. Figure 23b breaks down linearity and repeatability for each group. All three groups exhibit strong linearity, with 10CB and 15CB approaching 1.0. Repeatability, however, diverges sharply: 10CB leads at ~1.0, 05CB follows at ~0.66, and 15CB trails at only ~0.40. This shows that high CB loading preserves linearity but introduces agglomeration effects that severely compromise signal reversibility under cyclic loading. Figure 23c reports the failure early-warning amplitude, with the vertical axis representing FCR increment (%). The 05CB specimen produces an FCR increment of only ~100%, too low to generate a meaningful damage signal. Both 10CB and 15CB reach FCR increments of ~1750–1800%, each demonstrating strong electrical early-warning capability for incipient damage detection.

**Figure 24:**
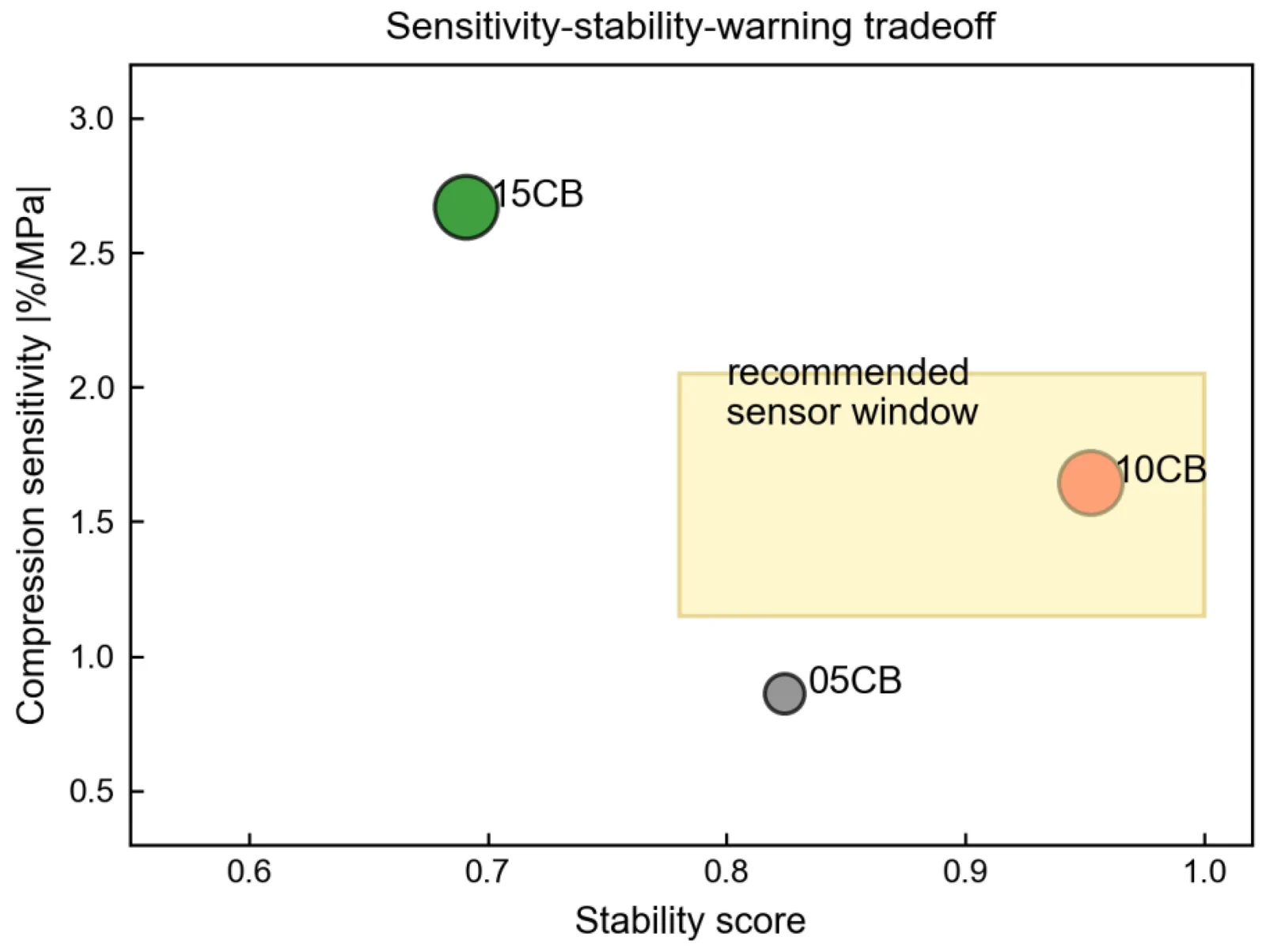
Two-dimensional performance evaluation: compressive sensitivity versus stability score, with the recommended sensor window.

To assess overall performance within a unified framework, Figure 24 plots stability against compressive sensitivity as a two-dimensional performance map. In terms of spatial distribution, 05CB occupies the high-stability, low-sensitivity region of the map. The 15CB data point sits at the highest compressive sensitivity (~2.7%/MPa), yet its stability score is the lowest of the three. The 10CB point falls within the recommended sensor window, the highlighted region representing the ideal engineering zone where both sensitivity and stability are satisfactory. Within this window, 10CB achieves a stability score of ~0.95 and a compressive sensitivity of ~1.6%/MPa, striking a practical balance between the two metrics. Figure 24 makes clear that each dosage occupies a distinct performance niche. The 10CB formulation offers the most balanced configuration; 15CB skews toward high sensitivity at the expense of stability; and 05CB is characterized primarily by limited sensitivity.

Figure 22, Figure 23 and Figure 24 together support a clear engineering selection guideline. For long-term structural monitoring of concrete, 10CB is the recommended formulation. Its signal reliability, repeatability, and environmental durability all meet the demands of extended service. Where the design objective is high-sensitivity failure warning at limit damage states, 15CB offers a viable high-sensitivity alternative. Its substantially reduced stability must, however, be carefully evaluated and compensated for in any practical deployment.

### 3.9. Proposed Mechanism Chain

**Figure 25:**
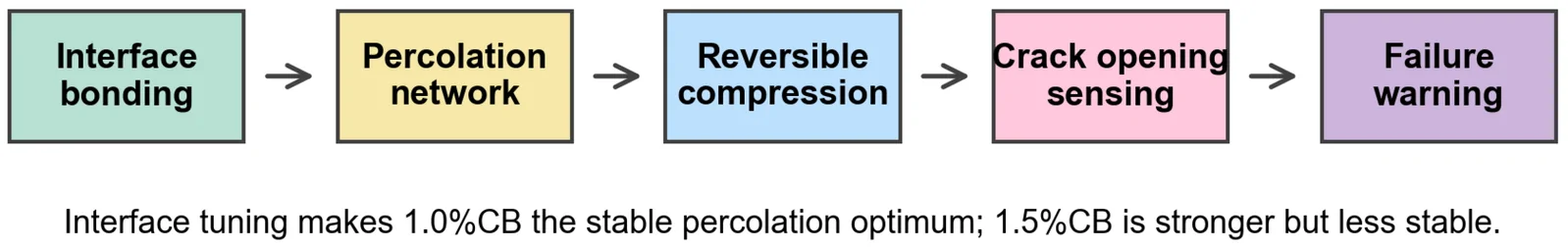
Schematic summary of the experimental workflow and interpretation framework for the CB/PP fiber cement-based sensor: interfacial characterization → conductive-network assessment → cyclic-compression response → crack-opening response → failure-warning evaluation.

Figure 25 provides a schematic summary of the experimental workflow used in this study to organize relationships among interfacial characterization, conductive-network assessment, and electromechanical response. The workflow is arranged in five analysis steps: interfacial characterization, conductive-network evaluation, reversible compression response, crack-opening response, and failure-warning evaluation. These steps reflect the sequence in which the experimental observations are analyzed and linked, rather than experimentally verified stages of a mechanistic pathway. Figure 25 is only intended as a conceptual illustration of this workflow and does not constitute independent experimental evidence for, or direct proof of, the proposed sensing mechanism. Accordingly, the role of the diagram is to clarify the study logic and the connections among the different experimental modules.

The mechanistic chain begins with interfacial bonding control. As shown by the SEM-EDS results and consistent with the absence of new bands in the FTIR spectra, interfacial engineering directs CB nanoparticles to adsorb physically and uniformly onto the PP fiber surface, forming an effective coating layer. This transforms the PP fiber from a conventional crack-bridging reinforcement into a multifunctional composite fiber with integrated electrical conductivity. CB-coated fibers distribute randomly throughout the cement matrix, spanning internal pores and potential crack planes. In doing so, they establish a continuous, fiber-dominated conductive network that serves as a stable percolation backbone for all subsequent electrical responses.

Mechanical loading and electrical measurements constitute the subsequent steps of the workflow. Cyclic compression tests are used to evaluate reversible piezoresistive behavior, whereas notched bending and splitting tensile tests, together with CMOD and DIC measurements, are used to compare FCR evolution with crack opening and strain localization. The reversible FCR variation under cyclic compression, the progressive FCR increase during crack development, and the rapid increase near final damage are observations derived from the corresponding experimental results presented in the preceding sections. Figure 25 only organizes these observations into a sequential interpretation. The arrows and conductive-pathway changes shown in the schematic should therefore not be regarded as direct experimental visualization of conductive-network evolution or pathway breakage.

Taken together, Figure 25 serves as a graphical overview of how the experimental modules and their corresponding observations are connected in the present study. The assessment that 10CB provides the most balanced sensing performance is based on the quantitative results presented in the preceding sections, including resistivity, sensitivity, repeatability, failure-warning response, and hygrothermal stability. Likewise, the higher sensitivity but lower stability observed for 15CB is supported by these measured performance indicators. Thus, Figure 25 should be regarded as a workflow and interpretation aid rather than as scientific evidence independently establishing a unified sensing mechanism. Its purpose is to summarize how the different experimental observations are considered together when interpreting the sensing behavior of the CB/PP fiber cementitious composites.

## 4. Conclusions

This study fabricated CB/PP fiber-embedded cementitious sensors at three CB dosages (0.5%, 1.0%, and 1.5%) using an interfacial engineering strategy. Microstructural characterization via SEM-EDS and FTIR was combined with synchronized electromechanical tests including cyclic compression, notched bending, and splitting tension. DIC strain field tracking and hygrothermal cycling durability tests were also incorporated. Together, these methods enabled a systematic investigation of conductive network formation, self-sensing behavior under multiple loading conditions, and environmental stability. The principal conclusions are as follows:

(1) Interfacial engineering promotes the formation of a uniform and stable CB coating on the PP fiber surface. CB nanoparticles anchor to the fiber surface through physical hydrophobic adsorption and van der Waals interaction; FTIR shows no new absorption bands and no shift in the PP backbone bands, indicating that no chemical bonding is formed at the CB–PP interface. This stable anchoring effect may facilitate hydration product accumulation around PP fibers, forming an interfacial structure consistent with heterogeneous C-S-H nucleation. At 1.0% CB, the interface index reaches its maximum, and the C/(Ca + Si) atomic ratio on the fiber surface drops from 2.1 in the 05CB group to 0.9. This transforms the PP fiber from a single-function crack-bridging component into a multifunctional conductive unit that integrates reinforcement and electrical sensing.
(2) CB dosage governs the percolation state of the conductive network and the accessible sensing performance window. The 05CB (0.5% CB) specimen remains below the percolation threshold, with a dry-state resistivity as high as 1.4 × 10^6^ Ω·cm and a discontinuous conductive network. The 10CB (1.0% CB) specimen falls within the effective percolation window (~0.9–1.2%), with resistivity dropping by nearly one order of magnitude to 2.5 × 10^5^ Ω·cm. The moisture stability index peaks while the agglomeration index remains low. The 15CB (1.5% CB) specimen reduces resistivity further to 1.4 × 10^5^ Ω·cm, yet the agglomeration index rises sharply once CB content exceeds 1.2%. The competition between the percolation curve and the two indices identifies 10CB as the inflection point at which the network transitions from disconnected to fully continuous. It represents the optimal balance between conductive connectivity and structural stability.
(3) Across cyclic compression, flexural cracking, and splitting tension, the 10CB formulation delivers the best overall performance in linearity, repeatability, and crack response stability. Under cyclic compression, 10CB achieves a repeatability exceeding 92%, substantially higher than the 15CB (<76%). Under flexural loading, 10CB yields a CMOD sensitivity of ~200%/mm with a smooth FCR–CMOD curve. The 05CB response is negligible, and 15CB produces a lower amplitude than 10CB due to network saturation. Under splitting tension, 10CB achieves a gauge factor GF ≈ 100 and an FCR–strain linearity of R^2^ > 0.92. It also exhibits a clear bilinear response characterized by the slope ratio (“S_2_/S_1_ ≈ 3.0”). This bilinear feature effectively distinguishes stable micro-crack propagation from macro-crack coalescence. Across all loading conditions, 10CB is the recommended formulation for long-term structural health monitoring.
(4) Under extreme loading, 15CB produces stronger failure-warning signals, with a slope ratio “S_2_/S_1_ ≈ 10” in the splitting softening stage and an FCR increment exceeding 1750%. This makes it attractive for high-sensitivity failure detection scenarios. Its linearity error exceeds 21% and repeatability falls below 76%. After hygrothermal cycling, resistance drift reaches ~23% and gauge factor retention drops to 83%, compared with 93% for 10CB. The combined degradation in hysteresis, drift, and environmental stability substantially limits its suitability as a long-term sensing material. DIC strain fields and the conductive pathway evolution model further reveal that the 15CB network is excessively dense. In the macro-crack stage, localized crack events trigger cascading pathway failures across a wide area, producing abrupt signal spikes that lack the stability required for reliable monitoring.
(5) The complete evidence chain established in this study—interfacial engineering, percolation window, electromechanical response, and damage warning—provides a mechanistically informed design framework for CB/PP fiber-reinforced cementitious sensors. At insufficient CB content, 05CB fails to form an effective conductive network. At excessive CB content, 15CB yields larger signal amplitudes but suffers from pronounced agglomeration and stability degradation. At the intermediate dosage, 10CB achieves the optimal balance among conductive connectivity, sensing linearity, response repeatability, and environmental durability. This interfacial engineering strategy offers a practical pathway for translating self-sensing cementitious materials from laboratory research into field deployment, balancing sensitivity with long-term reliability. This strategy holds particular promise for seismic damage assessment and post-earthquake safety diagnosis in critical infrastructure such as high-speed railway bridges.

## Data Availability

The original contributions presented in this study are included in the article. Further inquiries can be directed to the corresponding author.
